# Regulation of Early Host Immune Responses Shapes the Pathogenicity of Avian Influenza A Virus

**DOI:** 10.3389/fmicb.2019.02007

**Published:** 2019-09-11

**Authors:** Jiya Sun, Jingfeng Wang, Xuye Yuan, Xiangwei Wu, Tianqi Sui, Aiping Wu, Genhong Cheng, Taijiao Jiang

**Affiliations:** ^1^Suzhou Institute of Systems Medicine, Suzhou, China; ^2^Center for Systems Medicine, Institute of Basic Medical Sciences, Chinese Academy of Medical Sciences and Peking Union Medical College, Beijing, China; ^3^Department of Microbiology, Immunology and Molecular Genetics, University of California, Los Angeles, Los Angeles, CA, United States

**Keywords:** influenza, virus–host interaction, early immune response, gene network, interferon

## Abstract

Avian influenza A viruses (IAV) can cross the species barrier and cause disease in humans. Understanding the pathogenesis of avian IAV remains a challenge. Interferon-mediated antiviral responses and multiple cytokines production are important host cellular antiviral immunity against IAV infection. To elucidate the pathogenicity of avian IAV, a system approach was adopted to investigate dysregulation of the two host cellular antiviral immune responses in contrast with human IAV. As a result, we revealed that avian IAV not only disrupted normal early host cellular interferon-mediated antiviral responses, but also caused abnormal cytokines production through different pathways. For avian IAV infection, dysregulation of STAT2 was mainly responsible for abnormal cellular interferon-mediated antiviral responses, and IRF5 and NFKB1 played crucial roles in unusual cytokines production. In contrast, for human IAV infection, IRF1, IRF7, and STAT1 contributed to cellular cytokines production. Furthermore, differential activation of pattern recognition receptors (PRRs) likely led to avian IAV-related abnormal early host cellular antiviral immunity, where TLR7 and RIG-I were activated by avian and human IAV, respectively. Finally, a pathogenesis model was proposed that combined of early host cellular interferon-mediated antiviral responses with cytokines production could partly explain the pathogenicity of avian IAV. In conclusion, our study provides a new perspective of the pathogenesis of avian IAV, which will be helpful in preventing their infections in the future.

## Introduction

Influenza virus is a long-term threat to global public health. In contrast to human influenza A viruses (IAV) such as H1N1 (Du et al., 2017) that usually causes seasonal epidemic every year, avian IAV such as H5N1 (Creanga et al., 2017; Peng et al., 2017) and H7N9 (Wu et al., 2013) suddenly jump from their avian hosts to human and cause a high mortality rate, about 60% for H5N1 and 38% for H7N9 (Yu et al., 2008; Gao et al., 2013), which has brought serious social panic (Su et al., 2015). In order to improve the ability to control avian IAV, there is an urgent need for a deep understanding of their pathogenicities.

Computationally, the pathogenicity of avian IAV is often explored in two ways: identification of viral genome mutations and characterizing host cellular responses by using *in vitro* cell lines (Li et al., 2011; Josset et al., 2014; Simon et al., 2015) or *in vivo* mammalian models (Belser and Tumpey, 2013; Morrison et al., 2014; Su et al., 2017). To date, quite a few avian IAV specific genome mutations have been reported to confer binding to the human-type receptor (Auewarakul et al., 2007), increase replication efficiency in mammalian cells (Czudai-Matwich et al., 2014) and antagonize interferon production (Li et al., 2006). Many studies have taken a systematic approach to investigate virus-induced host cellular transcriptomes (Li et al., 2011; Josset et al., 2014; Simon et al., 2015; Chasman et al., 2016) for elucidation of avian IAV pathogenesis. For instance, Li et al. (2011) performed a co-expression network analysis of transcriptomes under H5N1 infection and identified that keratinization process was a potential novel regulator of its pathogenesis. Josset et al. (2014) revealed that H7N9 specifically elicited host cellular responses related to regulating cell cycle and gene transcription. Chasman et al. (2016) inferred pathogenicity-related gene modules by integrating cellular transcriptomes involving highly and low pathogenic IAV. Although these findings provide some clues to the pathogenicity of avian IAV in the context of the complicated virus–host interaction, dysregulation of early host cellular antiviral immune responses has not been systematically investigated.

Naturally, upon infection with IAV, host cells can recognize virus entry through the RIG-I signaling pathway (Loo and Gale, 2011), which leads to cellular immune responses including expression of antiviral response genes and production of multiple cytokines. Host cellular antiviral response genes are induced via activation of the type I interferon signaling pathway (Schneider et al., 2014; McNab et al., 2015), which is leveraged by host cells to build the first defense line against virus invasion. Cytokines are cell-to-cell signaling proteins that can activate immune cells. During lethal influenza virus infection, dysregulation of early induced cytokines is likely associated with mortality (Vogel et al., 2014). Gerlach et al. (2013) observed significant differences in early host cellular immune responses between seasonal and pandemic human IAV. Thus, it is reasonable to focus on early host cellular antiviral immune responses to decipher the pathogenesis of highly pathogenic avian IAV.

In this study, we systematically compared the two host cellular antiviral immune responses including interferon-meditated antiviral responses and cytokines production between avian IAV (H5N1 and H7N9) and human IAV (H1N1). Through focusing on a set of host cellular antiviral state genes (ASGs) and multiple cytokines, we proposed a novel unified model to explain the pathogenicity of highly pathogenic avian IAV, which resulted from dysregulation of early host cellular interferon-mediated antiviral responses and cytokines production.

## Materials and Methods

### Dataset Collection

Raw microarray data of high-quality Calu-3 cell transcriptomes treatment by interferon-alpha (IFN-α) (GSE70217), H1N1 (GSE80697 and GSE37571), H5N1 [GSE76599 and GSE28166 (Li et al., 2011)] or H7N9 (GSE69026) were downloaded from NCBI GEO database. The used wild-type influenza strains were A/California/04/2009 (H1N1), A/Vietnam/1203/2004 (H5N1), and A/Anhui/1/2013 (H7N9), respectively. Three mutant strains were H5N1-PB2-K627E, H5N1-NS1-trunc124 and H7N9-NS1-103F/106M. K627E mutation in PB2 as well as 103F and 106M mutations in NS1 reduce viral replication and virulence in mammalian cells (Dankar et al., 2011; Min et al., 2013). The 90-amino-acid truncation at the C-terminus of NS1 reduces the virus capacity to antagonize host cellular antiviral^1^ responses (Hale et al., 2008).

### Data Processing and DEGs Identification

Background correction and between-arrays normalization were performed using limma (Ritchie et al., 2015) package in R. Control probes and low expressed probes were removed. Based on the cutoff of the 95th percentile of negative control probes on each array, probes that were at least 10% brighter than the negative controls were considered as being well expressed. Probes that expressed at least 10% of all of the arrays were used. For multiple probes with the same gene annotation, the probe with maximum mean expression intensity was finally chosen. Gene expression intensity was transformed with log2 before further downstream analysis. The plotMDS method from the limma package was used to remove outlier samples. Compared with matched mock, the limma package was employed to identify DEGs [|log2(fold change, FC)|≥ 1, *p*-value ≤ 0.05].

### Clustering of ASGs

Based on the time-series transcriptomes under treatment by 500 U/ml IFN-α (GSE70217), the ASGs were first divided into eight clusters on the basis of their log2FC values (the breaks were −3, −2, −1, 0, 1, 2, and 3) at 6 h, and further subdivided into small clusters by hierarchical clustering, which was based on gene expression vectors with three elements comprised of log2FC at 6 h and differences of log2FC between adjacent time points. The hclust function in R was used to perform hierarchical clustering.

### Pathway Annotation

The five pathways related to cellular survival and death were from the KEGG pathway database.

### Transcription Factor Enrichment Analysis

Gene sets related to transcription factor binding motifs were downloaded from the Molecular Signatures Database^1^ (v6.1). Transcription factor enrichment analysis was based on Fisher’s exact test with 22810 human protein-coding genes as background. The fisher.test function in R was used to perform Fisher’s exact test.

### Construction of Regulatory Network of ASGs

The LASSO algorithm developed in the glmnet R package was used to infer gene regulatory network by performing regularized linear regression (LR) between each of 1658 ASGs and 1124 TFs. Human TF list was from the paper by Narang et al. (2015). Prediction accuracy was evaluated using Pearson’s correlation coefficient (PCC) between real and predicted gene expression levels. To assess influences of overfitting on built gene regulatory models, the LR model was used to predict expression levels of ASGs based on their assigned transcription factors (TFs) by LASSO models. The cor.test function in R was used to calculate PCC.

### Construction of IAV Strain-Specific Regulatory Network of Cytokines

First, the manually curated regulatory relationships between TFs and cytokine genes were downloaded from the CytReg database (Carrasco Pro et al., 2018). Then, the PCC between a pair of TF and cytokine gene was calculated using time-series transcriptomes, in which only differentially expressed TFs and cytokines were considered. Finally, all pairs of TF-cytokine interactions with PCCs of at least 0.7 were used to construct a virus strain-specific regulatory network of cytokines.

## Results

### Overview of Study

**FIGURE 1:**
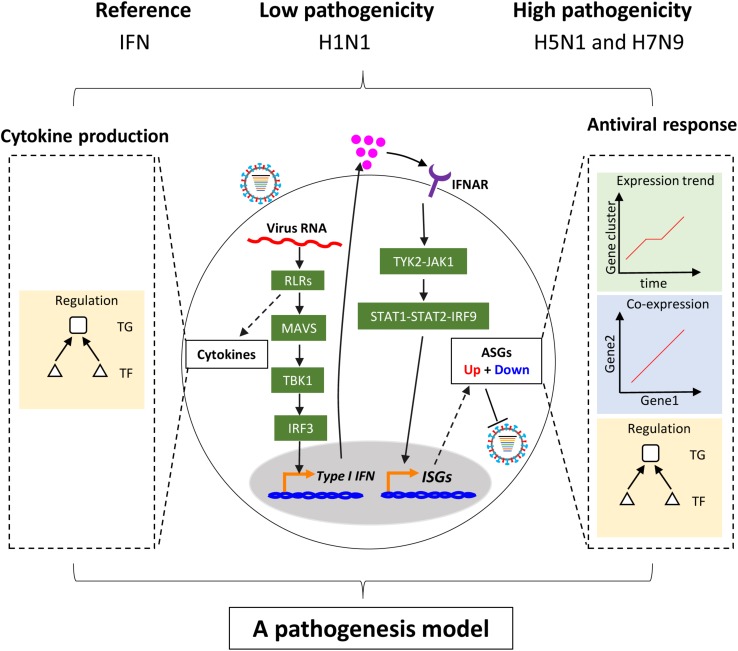
Schema of study. Upon infection of influenza virus, viral RNAs are rapidly recognized by the RIG-I-like receptor (RLR) of host cells. On the one hand, RLR-dependent pathway induces secretion of type I interferons. The type I interferons bind to their receptors and activate the canonical Jak-Stat pathway, which further induces the expression of hundreds of interferon-stimulated genes (ISGs). The ISGs enable host cells to establish antiviral state with thousands of upregulated or downregulated host cellular antiviral state genes (ASGs). On the other hand, Recognition of virus triggers production of multiple cytokines including type I interferons through complex pathways. In this study, regulation of cellular antiviral responses and cytokines production was comprehensively compared between low and highly pathogenic IAV.

In this study, we focused on regulation of host cellular type I interferon-mediated antiviral responses (hereafter the term host cellular antiviral responses specially referred to the interferon-mediated) and multiple cytokines production to understand the pathogenicity of highly pathogenic avian IAV (Figure 1). Overall, our analyses were comprised of three parts, in which the first was related to regulation of host cellular antiviral responses, the second for regulation of multiple cytokines production, and the last for generation and validation of a pathogenesis model of avian IAV. To make our results more reliable, three analysis groups related to IAV infections were designed (Supplementary Table S1). The first was the discovery group with datasets from the same laboratory, which were used to identify avian IAV strain-specific host cellular immune response patterns. The second was validation group with independent datasets from other laboratories, which were collected to validate the response patterns from the discovery group. The third was the mutation group with datasets involving avian IAV mutant strains from the same laboratory as the discovery group, which were utilized to further check the response patterns under wild-type avian IAV infections. The interferon dataset under IFN-α treatment was also from the same laboratory as the discovery group. For the three groups, the human or avian IAV strains had the same multiplicity of infection (MOI), in which the MOI of H1N1 was 3, and those of H7N9 and H5N1 were 1. All of the used transcriptomes were obtained using the same microarray platform to alleviate technical noise. In order to further avoid confusing inconsistencies due to different cell types, the Calu-3 cell line with many publicly available transcriptomes related to IAV infections were used. The Calu-3 cell is a human airway epithelial cell line from bronchial submucosal gland that is a major source of airway surface liquid, mucins, and other immunologically active substances in human lungs (Zhu et al., 2010). In addition, time points after H1N1 infection were no more than 48 h and those for H5N1 and H7N9 infections were within 24 h (Supplementary Table S1).

### Definition and Clustering of ASGs

**FIGURE 2:**
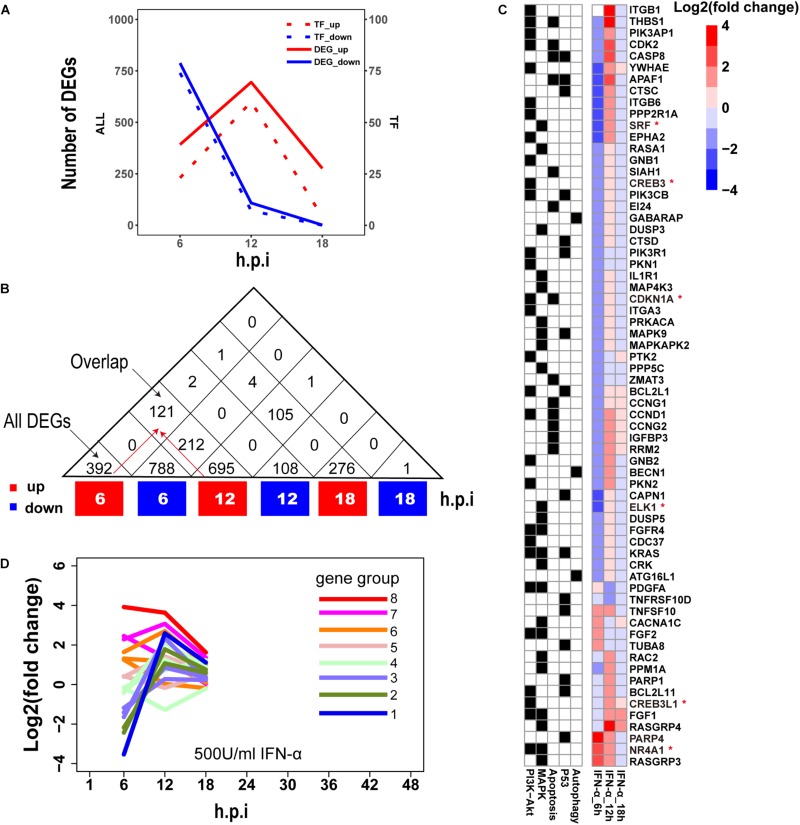
Characterization of ASGs under interferon treatment. **(A)** The number of interferon stimulated DEGs. The red and blue colors represent up- and downregulated DEGs, respectively. All (left) means all of DEGs and TF (right) for TF DEGs. **(B)** The number of overlap DEGs between two separate sets of DEGs. The numbers in each square cell mean overlap gene count and the numbers in triangle cell represent total of up- or downregulated DEG at indicated times. Please find an overlap gene count following red arrows. **(C)** Dynamic expression profiles of survival- and death-related ASGs. In the left, black color means existence of gene in corresponding pathways, and white for absence. In the right, each cell represents fold change of gene expression with log2 transformation. The abbreviations PI3K-Akt, MAPK, Apoptosis, P53 and Autophagy denote PI3K-Akt signaling pathway, MAPK signaling pathway, Apoptosis pathways, P53 signaling pathway, and regulation of autophagy, respectively. The genes with red star (^∗^) are TFs. **(D)** Expression trends of the 1819 ASGs under interferon treatment. Lines with different colors represent eight big gene groups, each of which is clustered into small gene clusters with the same color.

Establishment of interferon-mediated antiviral state provides a crucial initial line of host defense against virus invasion (Levy and Garcia-Sastre, 2001). To evaluate host cellular antiviral state, a set of ASGs was first defined using time-series transcriptomes under 500 U/ml IFN-α treatment (Supplementary Table S1). As a result, we identified 1819 ASGs that exhibited significant differential expression for at least one time point. In order to reasonably cluster these ASGs, we characterized their dynamic expressions under interferon treatment. Firstly, we found that the numbers of downregulated ASGs with the maximum at 6 h gradually decreased over the period of time, which was very different from those of upregulated ASGs that reached the maximum at 12 h (Figure 2A). Interestingly, the numbers of ASGs and significantly differentially expressed TFs were highly correlated (Figure 2A), which supported that transcription of ASGs was under control in a cascade manner due to interferon treatment alone. This fact was further confirmed by TF enrichment analysis, where upregulated ASGs were significantly enriched in IRF-related binding motifs at 6 and 12 h, while downregulated ASGs were dominated by other TFs such as SP1, ELK1, E4F1, ETS2, and SRF at 6 h (Supplementary Table S2). Secondly, downregulated ASGs showed time-specific expression dynamics, whereas many upregulated ASGs were still highly expressed at the next time point (Figure 2B). Compared with upregulated ASGs that rarely became downregulated at late infection stages, about 26.7% (212/788) of downregulated ASGs at 6 h changed into high expression at 12 h. Thirdly, we observed that the 66 ASGs from survival- and death-related signaling pathways exhibited dynamical expressions over times, where the majority of genes were downregulated and minor were upregulated at 6 h (Figure 2C). This was consistent with the fact that host cell can modulate cellular survival-death balance for its antiviral immunity (Upton and Chan, 2014).

Based on these observed time-specific expression features of the ASGs, we first divided the 1819 ASGs into eight gene groups on basis of their expression levels at the early time point (6 h), and then used gene expression changes between adjacent time points to further cluster each gene group into small gene clusters. Within each gene cluster, similar expression trends between genes demonstrated that our clustering approach had good performances (Supplementary Figure S1). In total, we identified 18 gene clusters that were prepared for evaluating the regulation of host cellular antiviral state during IAV infection (Supplementary Figure S1 and Supplementary Table S3). Moreover, the host cellular antiviral state established by IFN-α provided a reference to compare antiviral state changes during human and avian IAV infections (Figure 2D).

### Avian IAV Caused Distinctive Expression Trends of Early Response ASGs

**FIGURE 3:**
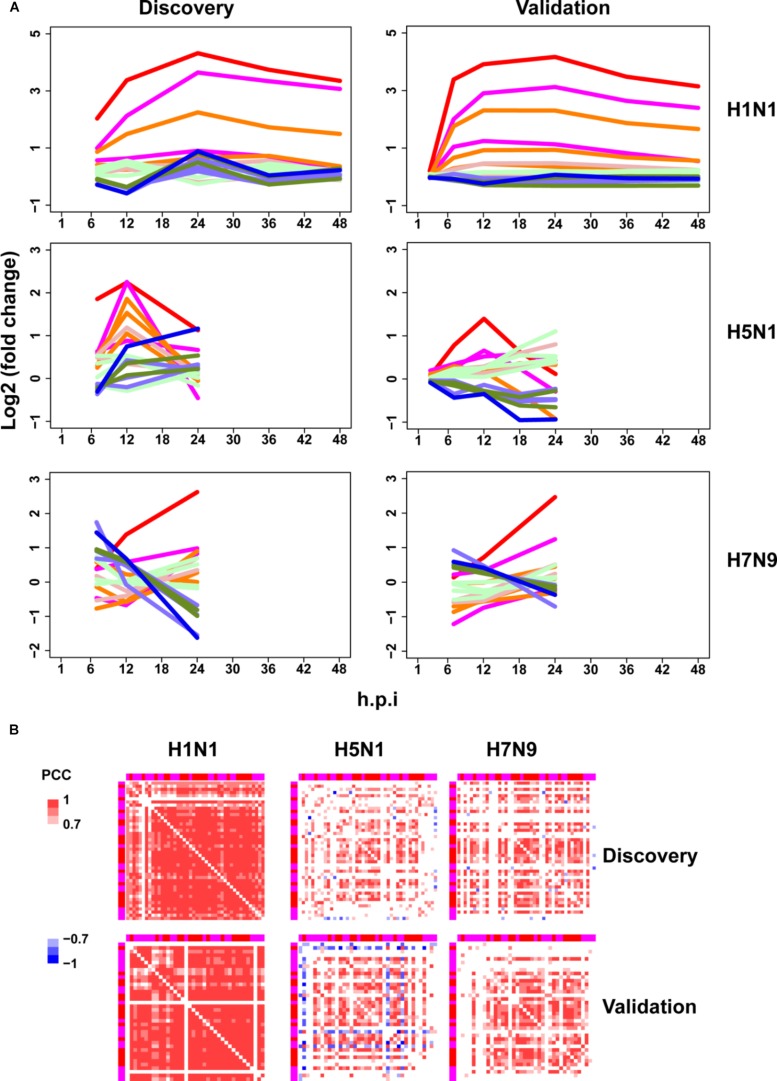
Perturbation of ASGs upon wild-type IAV infection. **(A)** Expression trends of the 1819 ASGs during infection of human and avian IAV. These lines have the same meanings as those of Figure 2D. For the same IAV strain, the discovery and validation datasets are from infection at the same MOI. **(B)** Expression correlations between the 44 early upregulated ASGs. The top and left annotation colors for different gene groups have the same meanings as those of lines in Figure 2D. In order to highlight strong correlations, highly positive correlations with PCCs ≥ 0.7 are set as red color, highly negative correlations with PCCs ≤ –0.7 for blue color, and weak correlations with PCCs > –0.7 and PCCs < 0.7 for white color.

Next, the above mentioned 18 gene clusters with rapid responses (early upregulated and early downregulated) or delayed responses (early silent or late-response) to IFN-α treatment were applied to investigation of host cellular antiviral state changes during human and avian IAV infections. As expected, we observed virus strain-specific dynamic regulation of cellular antiviral state in discovery datasets (Figure 3A left column). For low pathogenic human IAV H1N1, early upregulated ASGs were highly induced from the early to late infection stages, whereas early downregulated and late-response ASGs were suppressed. In contrast, highly pathogenic avian IAV H5N1 and H7N9 showed big differences. For H5N1, early upregulated ASGs were moderately induced in the early stage and remarkably suppressed in the late stage. Notably, the changes of cellular antiviral state induced by H7N9 were completely unexpected, in which early upregulated ASGs were initially suppressed and progressively became moderately activated, while early downregulated ASGs were initially activated and gradually suppressed. Although H5N1 and H7N9 are all avian IAV, our results strongly suggested that they had big differences in viral survival strategy. Furthermore, these findings were well supported in the independent validation datasets (Figure 3A right column).

### Synchronization of Early Upregulated ASGs Were Specifically Altered by Avian IAV

As stated above, highly pathogenic avian IAV caused distinctive time-specific gene expression trends of the ASGs. Besides, impaired gene synchronization between the ASGs should also be associated with the pathogenicity of avian IAV. To test this, we further investigated differential co-expression patterns of the ASGs between human and avian IAV. Here, gene co-expression was used to represent gene synchronization. According to the gene ordering from clustering of the ASGs (Supplementary Table S3), we visualized their pairwise PCC matrix (Supplementary Figure S2). After interferon treatment, it was obviously observed that early upregulated (from gene group 8 to 6) and early downregulated ASGs (from gene group 1 to 3) formed the most highly correlated modules, while early silent ASGs (from gene group 4 to 5) formed several moderately correlated modules (Supplementary Figure S2a). In contrast to early downregulated ASGs that most consisted of only one module, early upregulated ASGs corresponded to two clear modules, where one was from gene group 8 and 7 and the other from gene group 6. During human and avian IAV infections, the regular interferon-induced co-expression patterns of ASGs were widely perturbed but still indistinctly observed (Supplementary Figures S2b–d). Due to interferon treatment alone, the early downregulated ASGs was likely caused by the early downregulated ASGs, which was supported by TF enrichment analysis (Supplementary Table S2). Hence, we further only visualized the early upregulated group 8, 7, and 6, in which, interestingly, many ASGs showed conserved co-expression patterns between interferon treatment and H1N1 infection (Supplementary Figures S2e–h).

To further identify the consistent co-expression genes induced by interferon treatment and H1N1 infection, we clustered the PCC matrix of gene group 8 and 7. As a result, both of them exhibited a big co-expression gene module with 41 genes for interferon and 43 genes for H1N1 (Supplementary Figure S3). The two modules had 40 overlap genes, indicating their important roles in host cellular antiviral responses. Hence, co-expression of the 44 union genes from the two big gene clusters were further checked under avian IAV infections. Clearly, co-expression of the 44 early upregulated ASGs were significantly reduced by H5N1 and H7N9 (Figure 3B top). Furthermore, these findings were well validated by the independent validation datasets (Figure 3B bottom). Hence, avian IAV not only modulated the expression levels of early response ASGs, but also specifically disrupted the expression synchronization of early upregulated ASGs.

### Avian IAV Disrupted Interferon-Induced Normal Regulation of the 44 Early Upregulated ASGs

The finding that co-expression of the 44 early upregulated ASGs was made weaker by H5N1 and H7N9 (Figure 3B) likely resulted from impaired gene regulations caused by avian IAV. To verify this, we first employed the LASSO algorithm (Omranian et al., 2016) to build regulatory relationships between 1124 TFs and 1658 response ASGs in interferon treated cells, and then applied these gene regulatory models to predict expression levels of response ASGs in IAV infected cells. For these built gene regulatory models, the numbers of predicted TFs for response ASGs had an approximate normal distribution with the peak 12 (Supplementary Figure S4a). To examine influences of overfitting on the gene regulatory models, the widely used LR algorithm was adopted to predict expression levels of response ASGs in IAV infected cells, which was based on their assigned TFs by LASSO models. Overall, the LASSO and LR models showed very similar predictive powers for most response ASGs (Supplementary Figure S4b and Supplementary Table S5), indicating that the regulatory models of these response ASGs given by the LASSO algorithm were reliable.

**FIGURE 4:**
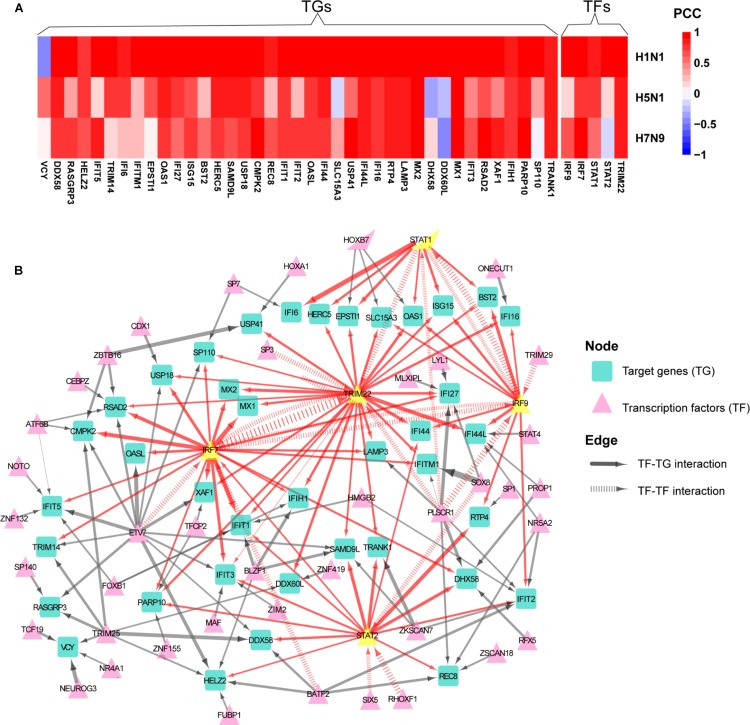
Aberrant regulation patterns of the 44 early upregulated ASGs upon infection of avian IAV. **(A)** Comparison of regulation patterns of the 44 ASGs during infection of human and avian IAV. High PCCs between true and predicted gene expression levels represent high prediction accuracies, which indicates that normal regulation patterns induced by interferon are kept by IAV, while low PCCs for disturbed regulation patterns. **(B)** Regulatory network of the 44 ASGs. For the 44 ASGs, their potential upstream regulators are given by the corresponding LASSO models with coefficients at least 0.1. The wider edges represent larger coefficients. The red edges are related to five key TFs with yellow color.

To determine whether normal gene regulation was disrupted by human or avian IAV, the PCCs between true and predicted expression levels were calculated, where high PCC indicated that the normal regulation induced by interferon was kept. Among the 44 early upregulated ASGs, we found that their regulation patterns were well maintained during H1N1 infection and more disrupted by avian IAV (Figure 4A). To further identify key TFs of the 44 ASGs, a meaningful regulatory network was built by selecting TFs with coefficients at least 0.1 in the LASSO models (Supplementary Table S4). Our regulatory network (Figure 4B) revealed that four TFs including IRF7, IRF9, STAT1, and STAT2 regulated most genes together or alone. In addition, TRIM22, a potential transcription factor, was predicted to contribute to expression levels of specific genes together with STAT1, STAT2, IRF9, and IRF7. For example, two TFs, including TRIM22 and IRF7, regulated MX1 and MX2, expression levels of which were accurately predicted in H1N1, H5N1, and H7N9 infected cells (Figure 4A and Supplementary Figure S5). Moreover, for H1N1, H5N1, and H7N9, expression levels of three TFs including STAT1, IRF7, and TRIM22 showed good prediction performances (Figure 4A and Supplementary Figure S5). On the contrary, STAT2 was not well predicted for avian IAV, indicating that dysregulation of STAT2 was most responsible for impaired co-expression patterns of the 44 genes. In addition, regulation of IRF9 was more disrupted by H5N1 than that of H7N9. In summary, our regulatory network explained impaired co-expression of the 44 genes and provided valuable insights into their potential regulators when binding motifs of many TFs were not available for now.

### Distinctive Regulation of Cytokines Production Between Human and Avian IAV

Thus far, the above results had demonstrated that highly pathogenic avian IAV can disrupt early host cellular antiviral responses in contrast to low pathogenic human IAV. In this work, host cellular antiviral responses were evaluated by the ASG genes that were defined by using transcriptomes under IFN-α treatment. During IAV infection, interferon, a type of cytokine, is usually rapidly induced. Although type I interferon can initiate host cellular antiviral responses, it actually induced few cytokines. In the dataset under IFN-α treatment (Supplementary Table S1), we observed that only 7 out of 113 human cytokines were induced with low expression levels. For severe influenza, complications or ultimately death are often associated with cytokine storm (Liu et al., 2016). Therefore, we further checked differences in regulation of multiple cytokines production between human and avian IAV.

For 113 human cytokines, there were 58, 50, and 30 cytokines with significant differential expressions for H1N1, H5N1, and H7N9, respectively, (Supplementary Figure S6), in which a few cytokines were strain-specific and many were shared by two or three IAV strains. Among these shared cytokines, we noticed that H5N1 always exhibited high expression levels while H1N1 and H7N9 showed low expression levels in the early infection stage (Supplementary Figure S6). However, we also observed that several cytokines were highly induced by H1N1 or H7N9 infections in the early stage. For example, CXCL10 and CCL5 were for H1N1 and CXCL5 for H7N9. Moreover, eight type I interferon genes including IFNB1, IFNA4, IFNA6, IFNA7, IFNA8, IFNA10, IFNA14, and IFN16 were more highly induced by H5N1 and repressed by H7N9 in the early stage. There are 13 human IFN-α subtypes, all utilizing a single type I IFN receptor (Gibbert et al., 2013). When treating human plasmacytoid dendritic cells by using various stimuli, Szubin et al. (2008) observed a rigid IFN-α subtype response pattern, in which each subtype was induced at similar relative levels for different stimuli. With respect to induction of ISGs, Moll et al. (2011) classified IFN-α subtypes into low, intermediate and high activity, which was confirmed by the protection of cells against influenza virus infection. Thus, regulating production of multiple types of type I interferons was crucial to the severity of IAV infection.

**FIGURE 5:**
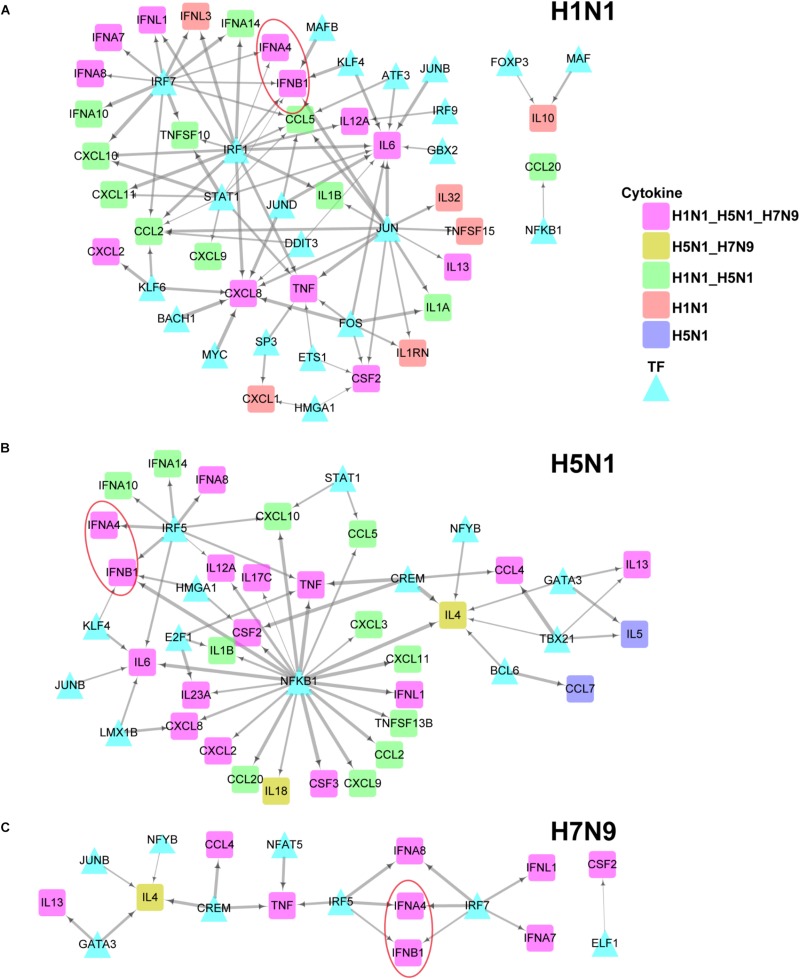
IAV strain-specific regulatory network of cytokines. **(A–C)** H1N1, H5N1 and H7N9 specific cytokine transcriptional regulation network. The different node colors represent differentially expressed cytokines specific for one strain or shared by two or more strains. The wider edges represent larger PCCs between a pair of TF and cytokine gene.

To further explore differences in regulation of these differentially expressed cytokines between human and avian IAV infections, we built their strain-specific transcriptional regulatory networks. Surprisingly, it was observed that human and avian IAV took very different approaches to control cytokines production. For H1N1, three regulators including IRF1, IRF7, and STAT1 played critical roles in regulation of cytokines production (Figure 5A). In contrast, H5N1 showed distinctive regulatory pathways, where IRF5 and NFKB1 were responsible for regulation of most cytokines including IFNB1 and IFNA4 (Figure 5B). Notably, for H7N9, IRF5 together with IRF7, contributed to regulation of IFNB1 and IFNA4 despite a small network available due to moderate host cellular immune responses (Figure 5C). These results demonstrated that dysregulation of multiple cytokines production during avian IAV infection arose from activation of completely different signaling pathways in contrast to human IAV.

### A Pathogenesis Model of Avian IAV

**FIGURE 6:**
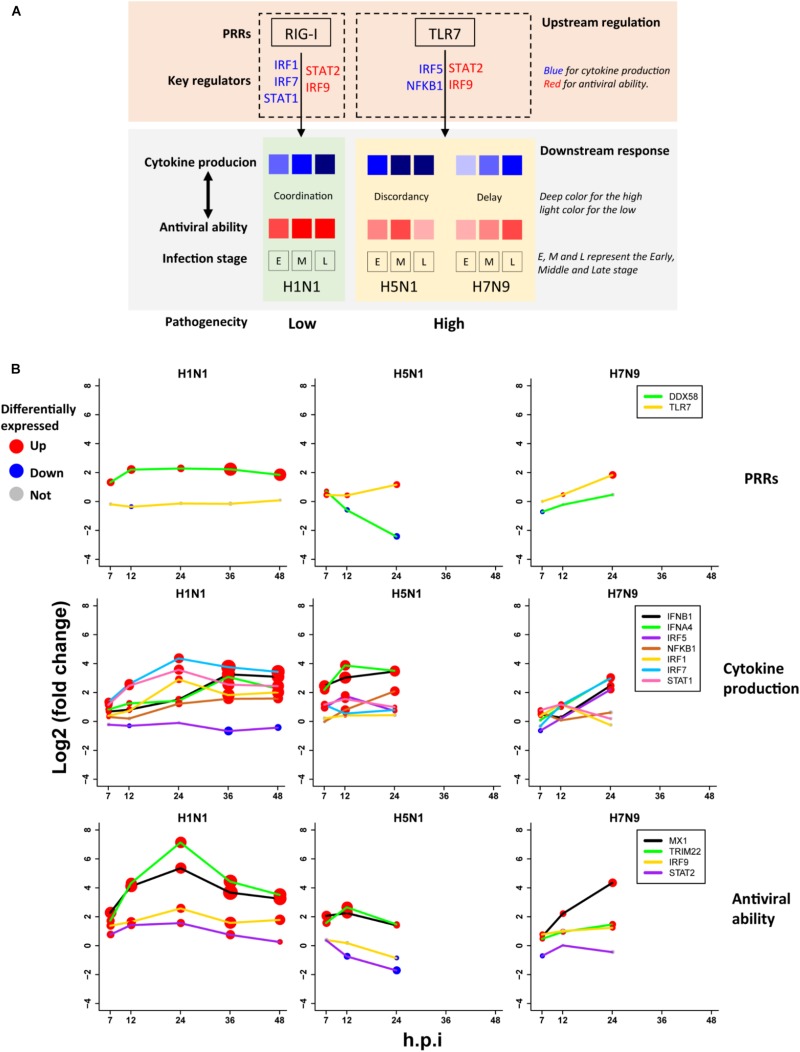
The pathogenesis model of avian IAV **(A)** A representation of the proposed pathogenesis model. **(B)** Dynamic expression levels of key genes involved in the pathogenesis model. The node size represents significance of gene differential expression, and the node color represents up-regulation (red), down-regulation (blue) or not significant (gray).

Based on the above findings, we proposed a unified model (Figure 6A) for explanation of avian IAV pathogenesis, which was centered on cooperation of the upstream interferon production (denoted by U) and the downstream interferon-induced antiviral responses (denoted by D). For H1N1, the low pathogenicity arose from high cooperation between the U and D with early moderate and late-high responses. However, two highly pathogenic avian IAV strains showed big differences. For H5N1, the conflict between the high U and low D over times caused the high pathogenicity. In contrast, H7N9 exhibited delayed but cooperative features between the U and D, in which the interferon production and antiviral responses were suppressed in the early stage, but remarkably increased in the late stage. These strain-specific patterns between the U and D were clearly seen from the dynamic patterns of interferon production represented by IFNB1 and IFNA4 (Figure 6B middle row), and antiviral responses represented by MX1 and TRIM22 (Figure 6B bottom row). For interferon production and antiviral responses, their dynamic expression levels were in line with those of their corresponding key TF regulators (Figures 6A,B).

Most importantly, it was observed that the key TF IRF1 that regulated H1N1-induced cytokines production was activated by H1N1 but suppressed by H5N1 and H7N9, while the key TF IRF5 for regulation of cytokines production during infection of avian IAV was activated by H5N1 and H7N9 but suppressed by H1N1 (Figure 6B middle row). The mutually exclusive expression of IRF1 and IRF5 prompted us to infer the underlying reasons. Relying on literature search, we found that TLR7, a type of pattern recognition receptor (PRR) for recognizing single strand RNA virus, can activate IRF5, which further induces type I interferon production (Schoenemeyer et al., 2005) and culminates in the activation of the transcription factor NF-KB that controls the expression of an array of inflammatory cytokines (Kawai and Akira, 2007). Interestingly, the expression of TLR7 was activated by H5N1 and H7N9 but suppressed by H1N1, while the canonical PRR RIG-I (also known as DDX58) was suppressed by H5N1 and H7N9 but activated by H1N1 (Figure 6B top row). The distinct usages of virus recognition receptors provided a solid support for our pathogenesis model.

### Validation of the Pathogenesis Model

**FIGURE 7:**
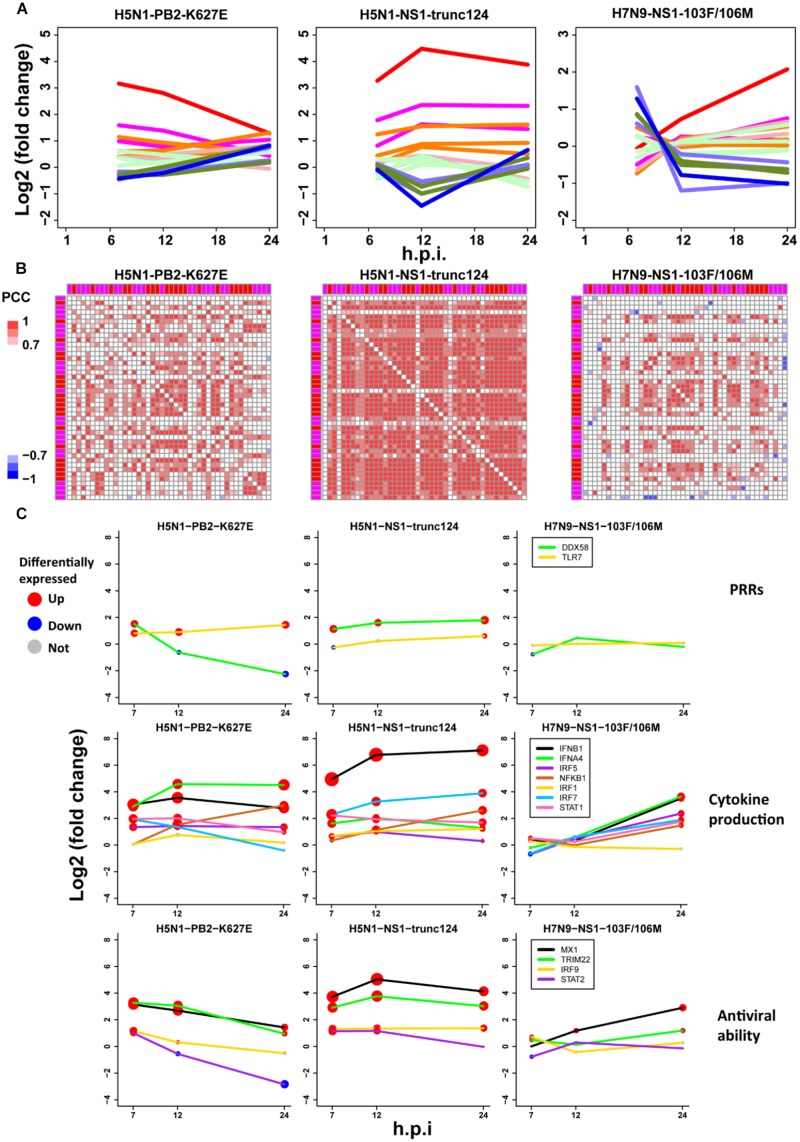
Validation of the pathogenesis model using avian IAV mutants. **(A)** Expression trends of all ASGs. The line colors have the same meanings as those of Figure 2D. **(B)** Expression correlations between the 44 early upregulated ASGs. The annotation colors and gene order are the same as those of Figure 3B. **(C)** Dynamic expression levels of key genes involving the pathogenesis model. The node size represents significance of gene differential expression, and the node color represents up-regulation (red), down-regulation (blue) or not significant (gray).

To further check the proposed pathogenesis model, we used transcriptomes under infection of low pathogenic avian IAV mutants. If the aberrant regulation of host cellular antiviral responses and cytokines production induced by wild-type avian IAV can be rescued, the pathogenesis model will be more reliable. Here, we focused on three mutants including H5N1-PB2-K627E, H5N1-NS1-trunc124, and H7N9-NS1-103F/106M. For H5N1-PB2-K627E, the gene expression trends of the whole ASGs became very similar to those under 500 U/ml IFN-α treatment (Figures 2A, 7D), which likely resulted from decreased viral replication in mammal cells due to the avian specific mutation PB2-K627E (Subbarao et al., 1993). However, the co-expression pattern of the 44 ASGs was still disrupted (Figures 3B, 7B). Furthermore, we observed that dynamic expression levels of two PRRs RIG-I and TLR7, key regulators of cytokines production such as IRF5, and antiviral responses related regulators such as STAT2 and IRF9 were still similar to those of wild-type H5N1 (Figure 7C). These evidences strongly supported that dysregulation of host cellular antiviral responses and cytokines production during H5N1 infection mainly arose from the nature of virus itself because decreased viral replication still caused their dysregulation. For H5N1-NS1-trunc124 that lost the ability of blocking IFN-β production (Qian et al., 2017), it was observed that the gene expression trends of the whole ASGs became very similar to those of H1N1 (Figures 3A, 7A) and the co-expression pattern of the 44 ASGs was rescued (Figures 3B, 7B). Actually, production of IFN-β was highly induced by the H5N1-NS1-trunc124 mutant. Interestingly, we also observed activation of RIG-I, repression of TLR7, and activation of IRF1, STAT2, and IRF9 during H5N1-NS1-trunc124 infection (Figure 7C). These observations were consistent with the fact that viral protein NS1 played a critical role in H5N1 pathogenicity (Zhou et al., 2010). Different from the two H5N1 mutants, the H7N9 mutant H7N9-NS1-103F/106M cannot rescue the gene expression trends of the whole ASGs (Figure 7A) and the co-expression pattern of the 44 ASGs (Figure 7B). During H7N9-NS1-103F/106M infection, however, we observed that TLR7 became repressed although RIG-I and key regulators of cytokines production and antiviral responses were not rescued (Figure 7C). These unexpected results suggested that this pair of NS1 mutations contributed less to H7N9 pathogenicity. Taken together, the data from the mutated avian IAV strains demonstrated the rationality of our proposed pathogenesis model.

## Discussion

In this study, we proposed a pathogenesis model of avian IAV by focusing on regulation of host cellular antiviral responses and cytokines production. In contrast to H1N1, both H5N1, and H7N9 disrupted normal early host cellular antiviral responses and cytokines production. However, time-specific cooperative patterns of cytokines production and early host cellular antiviral responses were very different between H5N1 and H7N9. These findings were based on the Calu-3 cell from bronchial submucosal gland that, in humans, is preferentially attached by avian IAV, such as H5N1, than by human IAV (van Riel et al., 2007). Therefore, protection of bronchial submucosal gland will be an effective approach to prevent infection of highly pathogenic avian IAV.

Despite different MOIs with 3 for H1N1 and 1 for H5N1 and H7N9, it was believed that differences of host cellular antiviral responses and cytokines production between human and avian IAV indeed arose from the nature of virus itself. The reasons were as follows: (1) The canonical PRR RIG-I was activated by H1N1 and repressed by H5N1 and H7N9, and the PRR TLR7 potentially recognizing H5N1 and H7N9 was repressed by H1N1. (2) For the same MOI, the H5N1-NS1-trunc124 mutant can activate the expression of RIG-I and inhibit the expression of TLR7. (3) Disruption of early host cellular antiviral responses during avian IAV infections was likely caused by dysregulation of STAT2, which can be rescued by the H5N1-NS1-trunc1241 mutant. (3) The differences of controlling host cellular early response antiviral genes between human and avian IAV were from not only gene expression levels (Figure 3A), but also gene co-expression (Figure 3B) that represented gene synchronization and was robust to various MOIs. (4) While the H5N1-PB2-K627E mutant with limited replication efficiency in mammalian cells induced normal antiviral gene expression trends but impaired gene co-expression patterns (Figures 7A,B), the H5N1-NS1-trunc124 mutant with decreased efficiency of antagonizing IFN-β production led to normal antiviral gene expression trends and gene co-expression patterns (Figures 7A,B). (5) Regulation of cytokines production was likely through completely different pathways during human and avian IAV infections (Figure 5). (6) Importantly, when infecting *in vitro* cells, H5N1 and H7N9 viruses caused infection 3–6 times faster than H1N1 virus (Simon et al., 2016). Hence, high MOI for H1N1 and low MOI for H5N1 and H7N9 were fair on assessing host cellular responses *in vitro*. All of these evidences demonstrated that specific host cellular antiviral immune responses to avian IAV likely resulted from inherent properties of virus.

For influenza virus, survival in host cell has been just like a battle of fighting for limited resources, in which virus uses the fewer to defeat the many. Our results implied possible survival strategies of avian IAV that modulated early host cellular antiviral responses. Actually, expression dynamics of most late-response ASGs were caused by early response ASGs in interferon treated cells (Figures 2A,B and Supplementary Table S2). Hence, for viruses, attacking key early response genes seems an effective approach to dominate host cells. Although the used human IAV H1N1 (A/California/04/2009), a major cause of seasonal influenza nowadays, once caused a pandemic, its pathogenicity is still much lower than avian IAV H5N1 and H7N9 (Morrison et al., 2014). Nevertheless, our results could not clearly explain the pathogenicity of the pandemic H1N1.

Generally, the RIG-I signaling cascade was used to trigger host cellular innate immunity against IAV infection (Liu et al., 2015). However, our results revealed big differences between human and avian IAV in triggering PRRs, where RIG-I was activated by H1N1 but suppressed by H5N1 and H7N9. A recent study by Jorgensen et al. (2018) reported that a serve influenza patient with defective RIG-I exhibited decreased antiviral responses as well as increased pro-inflammatory responses, which not only demonstrated that RIG-I played a critical role in host cellular responses to human IAV, but also supported that cooperation of host cellular antiviral responses and inflammatory responses was crucial for the pathogenesis of IAV. Although H5N1 and H7N9 blocked activation of RIG-I, they actually activated expression of TLR7, which is an important membrane-bound receptor triggered by single-stranded RNA and implicated in response to influenza virus. Wei et al. (2013) revealed that the TLR7 was involved in the early stage of antiviral innate immune responses in geese during infection of highly pathogenic H5N1. Thus, the severity in humans caused by H5N1 and H7N9 was very likely attributed to activation of the TLR7 pathway, which was normal in birds but not in humans.

Consistent with IAV classification by HA and NA groups (Nobusawa et al., 1991), differential host cellular antiviral responses revealed that H5N1 was more similar to H1N1 than to H7N9 (Figure 3A). Complementary to HA imprinting accounting for age biases of observed human cases between H5N1 and H7N9 (Gostic et al., 2016), our results also showed that expression patterns of early host cellular antiviral genes could explain these biases. For older adults favored by H7N9, their decreased immunity (Lee et al., 2017) cannot resist the early silent but late-high antiviral responses in host cells (Figure 2). However, decreased immune responses in older adults may protect them from H5N1 infection, which causes cytokine storm (Li et al., 2018) in young adults with the help of their strong immunity.

Unlike well-adapted human IAV that causes massive morbidity every year, avian IAV suddenly infect human with increased pathogenicity. In contrast to H5N1, H7N9 exhibits strange patterns of host cellular antiviral responses and cytokines production. Actually, our data supported that these likely arose from the nature of H7N9 virus because H7N9 not only suppressed the early upregulated ASGs but also activated early downregulated ASGs in the very early stage. As the normal cellular antiviral state established by IFN-α treatment (Figure 2D) indicated that high expression of early upregulated ASGs together with low expression of early downregulated ASGs were helpful for host cellular defense against virus, H7N9 likely had evolved to inhibit early host cellular antiviral responses for its replication. Since H7N9 was identified in March 2013, it has caused five epidemic waves in China. Due to its evolutionary genotypes (Ding et al., 2017), H7N9 may induce wave-specific regulation of host cellular antiviral responses. So, the identified 44 early upregulated ASGs may have potentials to evaluate evolution of H7N9 for monitoring host adaptation.

## Conclusion

In conclusion, our study provides a new perspective of the pathogenicity of highly pathogenic avian IAV that results from dysregulation of early host cellular antiviral responses and cytokines production, which will be helpful for prevention of avian IAV infection in the future.

## Data Availability

Publicly available datasets were analyzed in this study. This data can be found here: https://www.ncbi.nlm.nih.gov/geo/.

## Author Contributions

JS, JW, AW, GC, and TJ conceived and designed the experiments. XY contributed to the data collection. JS and AW contributed to the network construction. JS, JW, XW, and TS built the pathogenesis model. JS analyzed the data. JS and TJ wrote the manuscript. TJ, GC, and AW reviewed the manuscript.

## Conflict of Interest Statement

The authors declare that the research was conducted in the absence of any commercial or financial relationships that could be construed as a potential conflict of interest.

## Footnotes

1 http://software.broadinstitute.org/gsea/msigdb/index.jsp
