# Supplementary material for: Regulation of Early Host Immune Responses Shapes the Pathogenicity of Avian Influenza A Virus
Source: Front Microbiol. 2019 Sep 11;10:2007. doi: 10.3389/fmicb.2019.02007 (PMC6749051; doi:10.3389/fmicb.2019.02007)
Supplement: Supplementary file 1 [file Presentation_1.PPTX]

## Slide 1
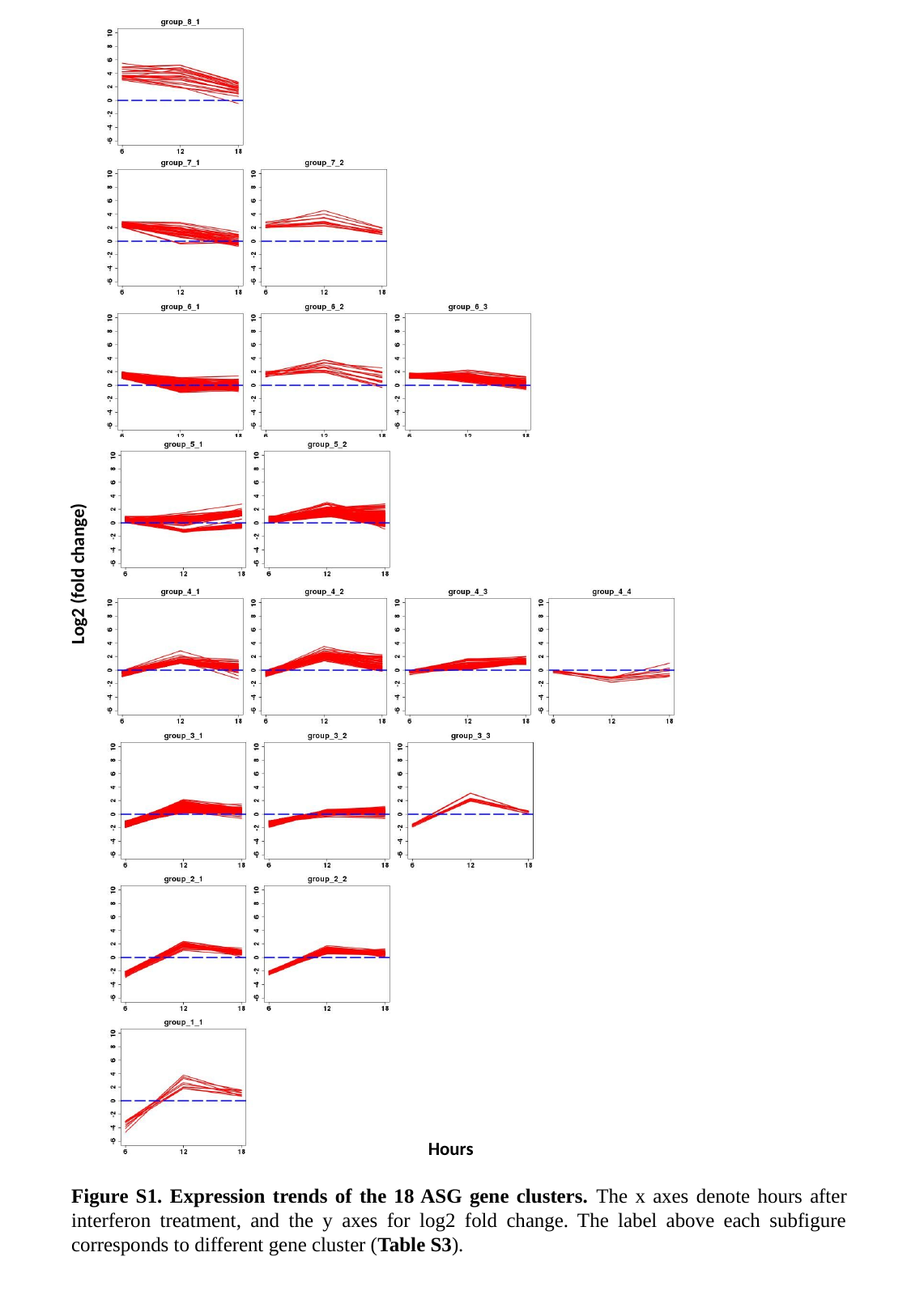

Log2 (fold change)
Hours
Figure S1. Expression trends of the 18 ASG gene clusters. The x axes denote hours after interferon treatment, and the y axes for log2 fold change. The label above each subfigure corresponds to different gene cluster (Table S3).

## Slide 2
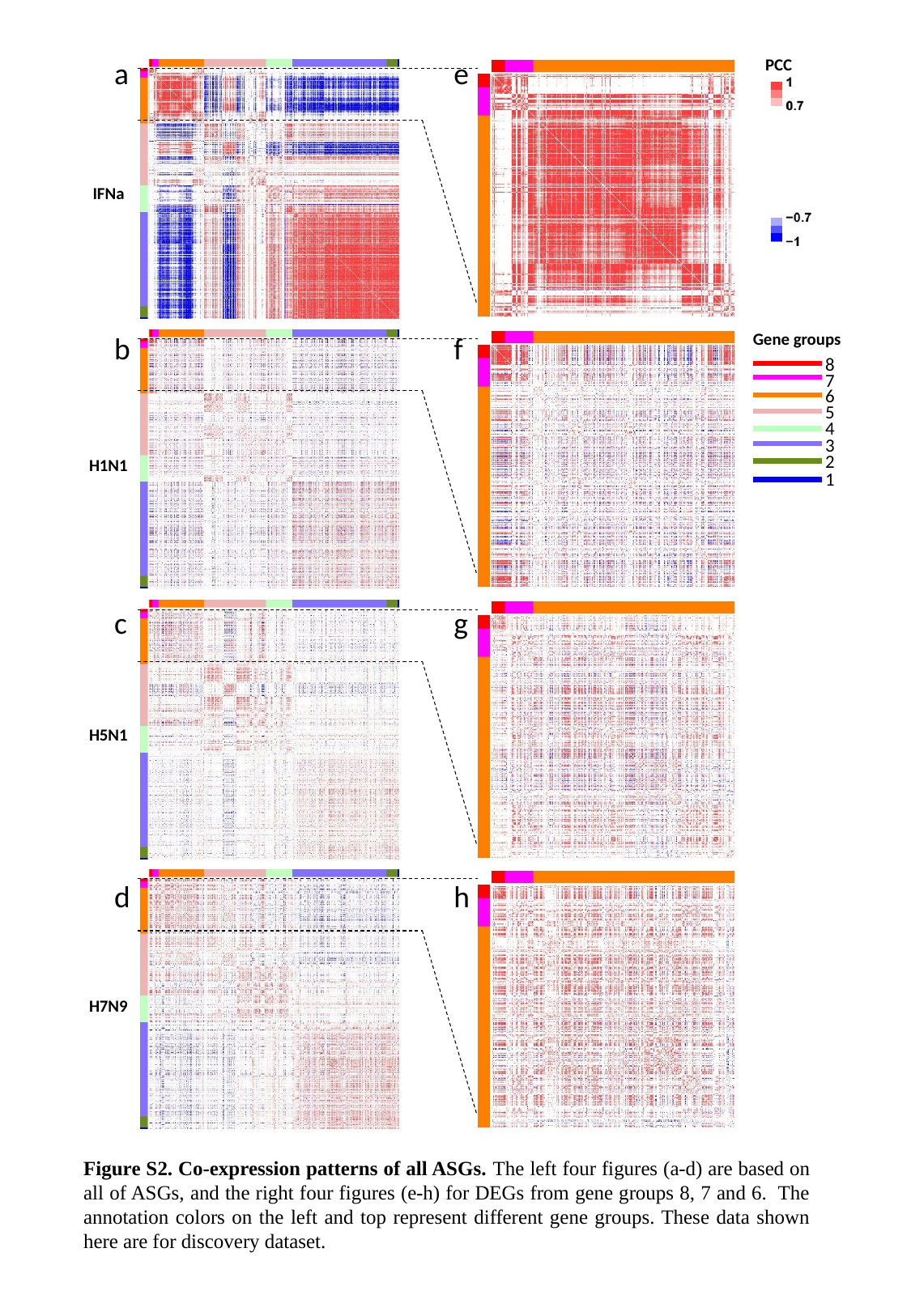

PCC
a
e
IFNa
Gene groups
b
f
8
7
6
5
4
3
2
H1N1
1
c
g
H5N1
d
h
H7N9
Figure S2. Co-expression patterns of all ASGs. The left four figures (a-d) are based on all of ASGs, and the right four figures (e-h) for DEGs from gene groups 8, 7 and 6. The annotation colors on the left and top represent different gene groups. These data shown here are for discovery dataset.

## Slide 3
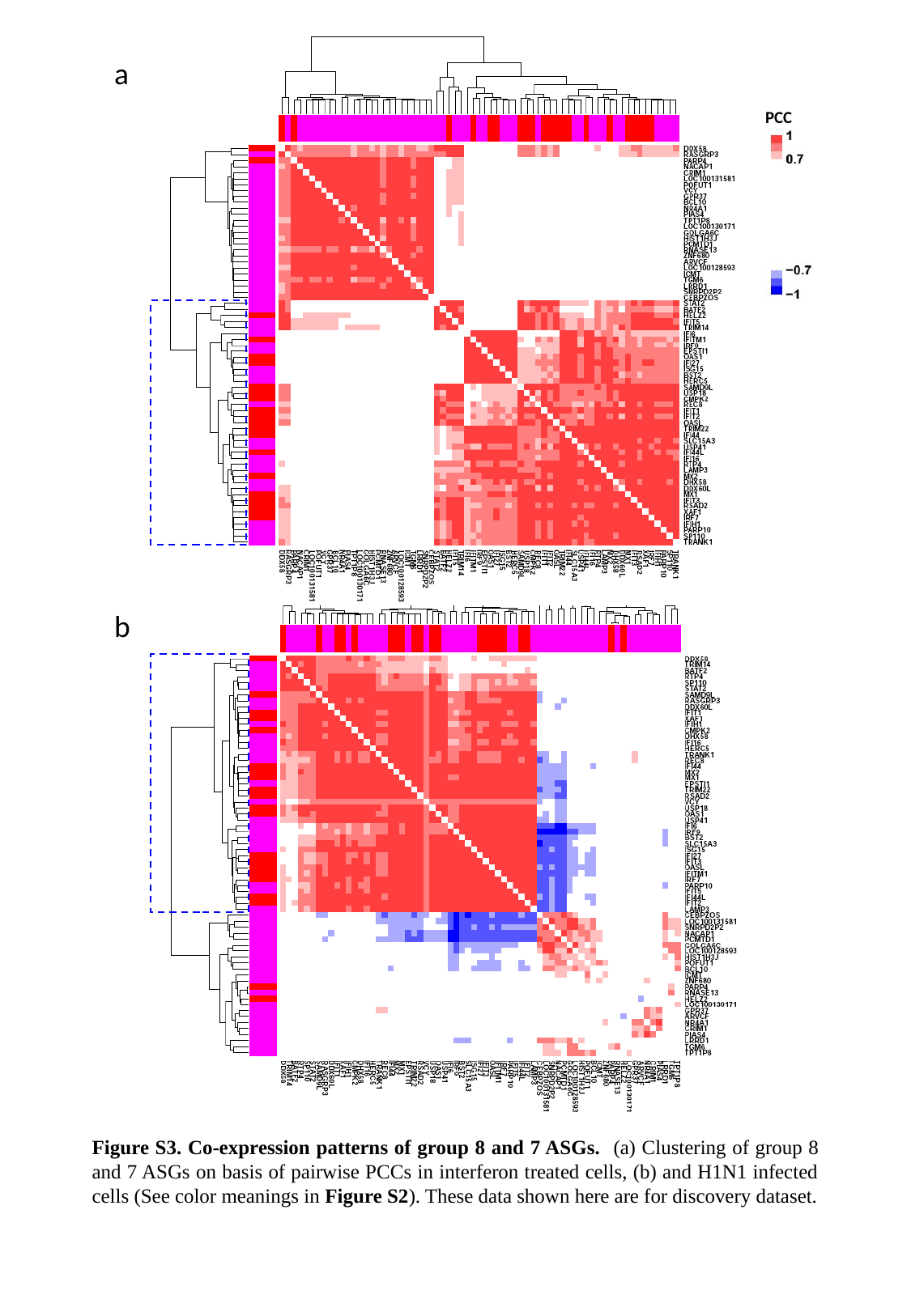

a
PCC
b
Figure S3. Co-expression patterns of group 8 and 7 ASGs. (a) Clustering of group 8 and 7 ASGs on basis of pairwise PCCs in interferon treated cells, (b) and H1N1 infected cells (See color meanings in Figure S2). These data shown here are for discovery dataset.

## Slide 4
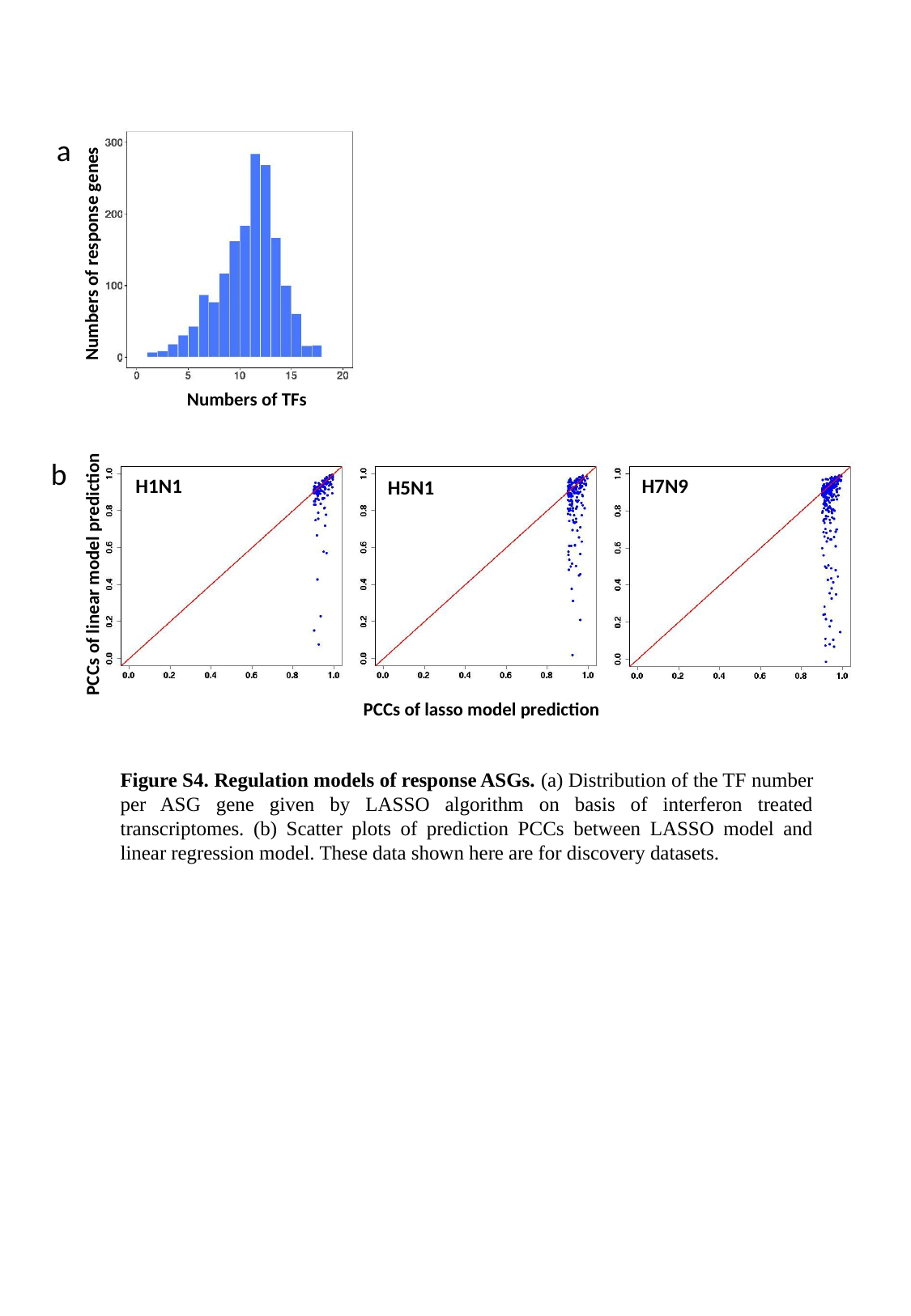

a
Numbers of response genes
Numbers of TFs
b
H1N1
H7N9
H5N1
PCCs of linear model prediction
PCCs of lasso model prediction
Figure S4. Regulation models of response ASGs. (a) Distribution of the TF number per ASG gene given by LASSO algorithm on basis of interferon treated transcriptomes. (b) Scatter plots of prediction PCCs between LASSO model and linear regression model. These data shown here are for discovery datasets.

## Slide 5
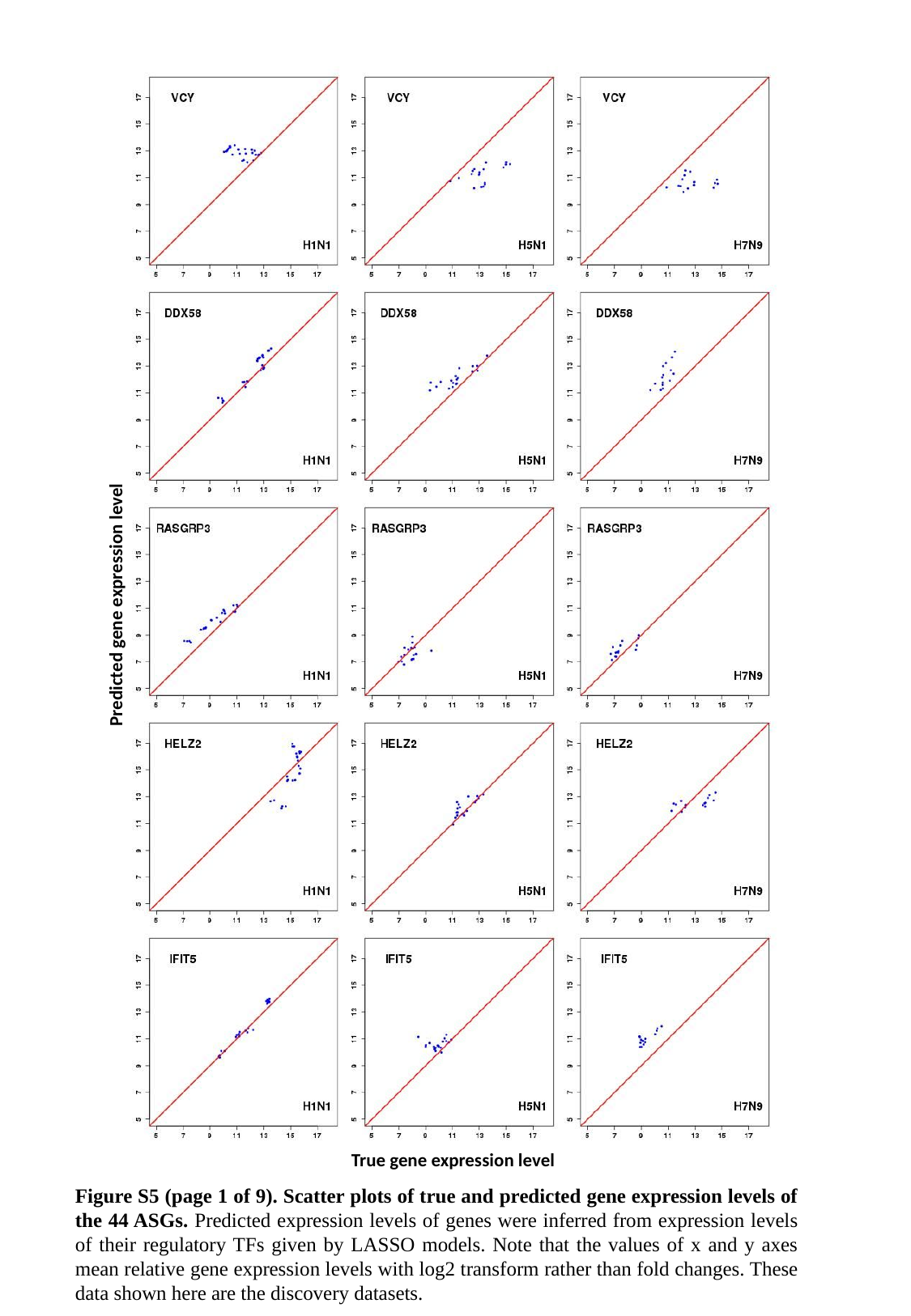

Predicted gene expression level
True gene expression level
Figure S5 (page 1 of 9). Scatter plots of true and predicted gene expression levels of the 44 ASGs. Predicted expression levels of genes were inferred from expression levels of their regulatory TFs given by LASSO models. Note that the values of x and y axes mean relative gene expression levels with log2 transform rather than fold changes. These data shown here are the discovery datasets.

## Slide 6
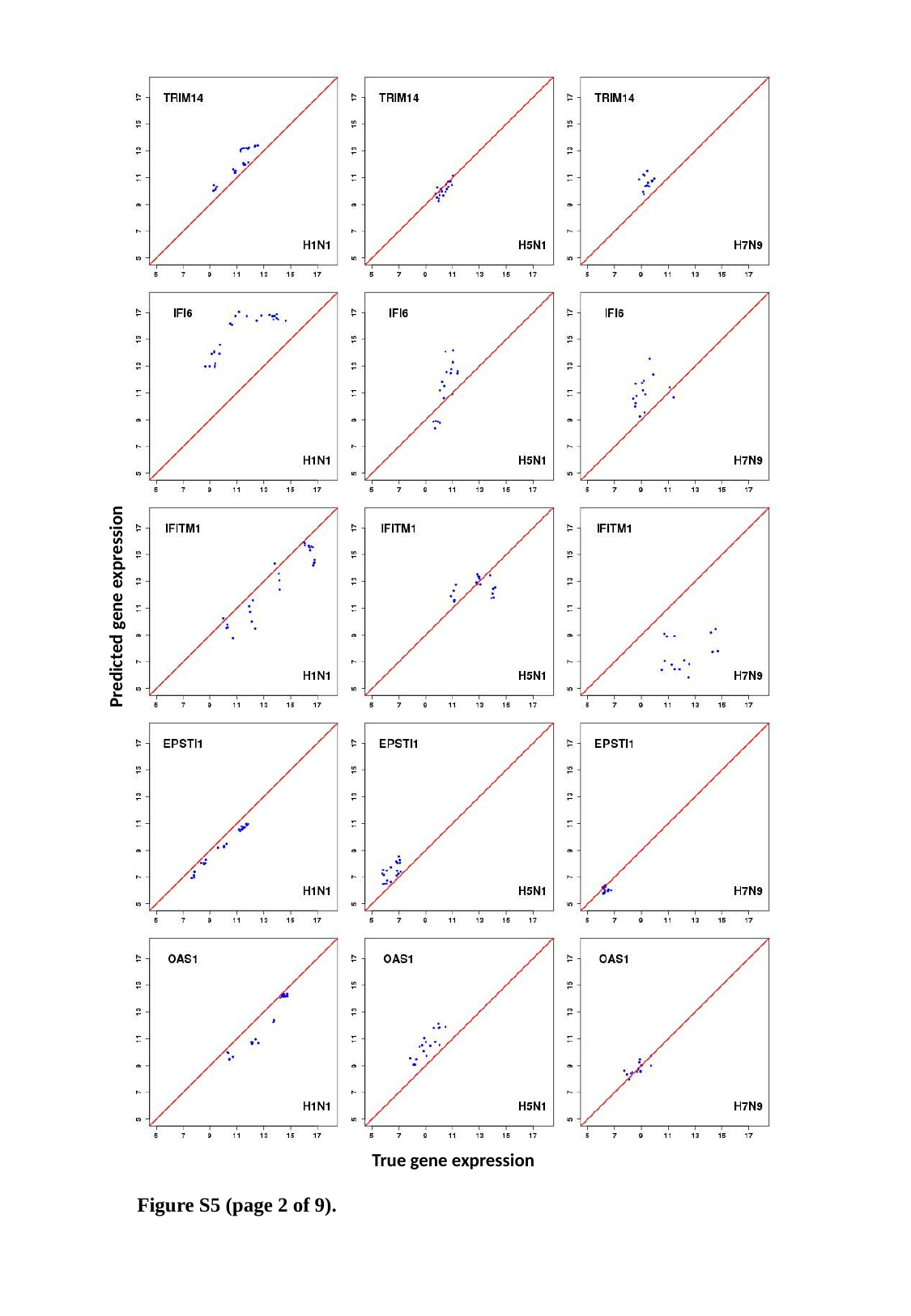

Predicted gene expression
True gene expression
Figure S5 (page 2 of 9).

## Slide 7
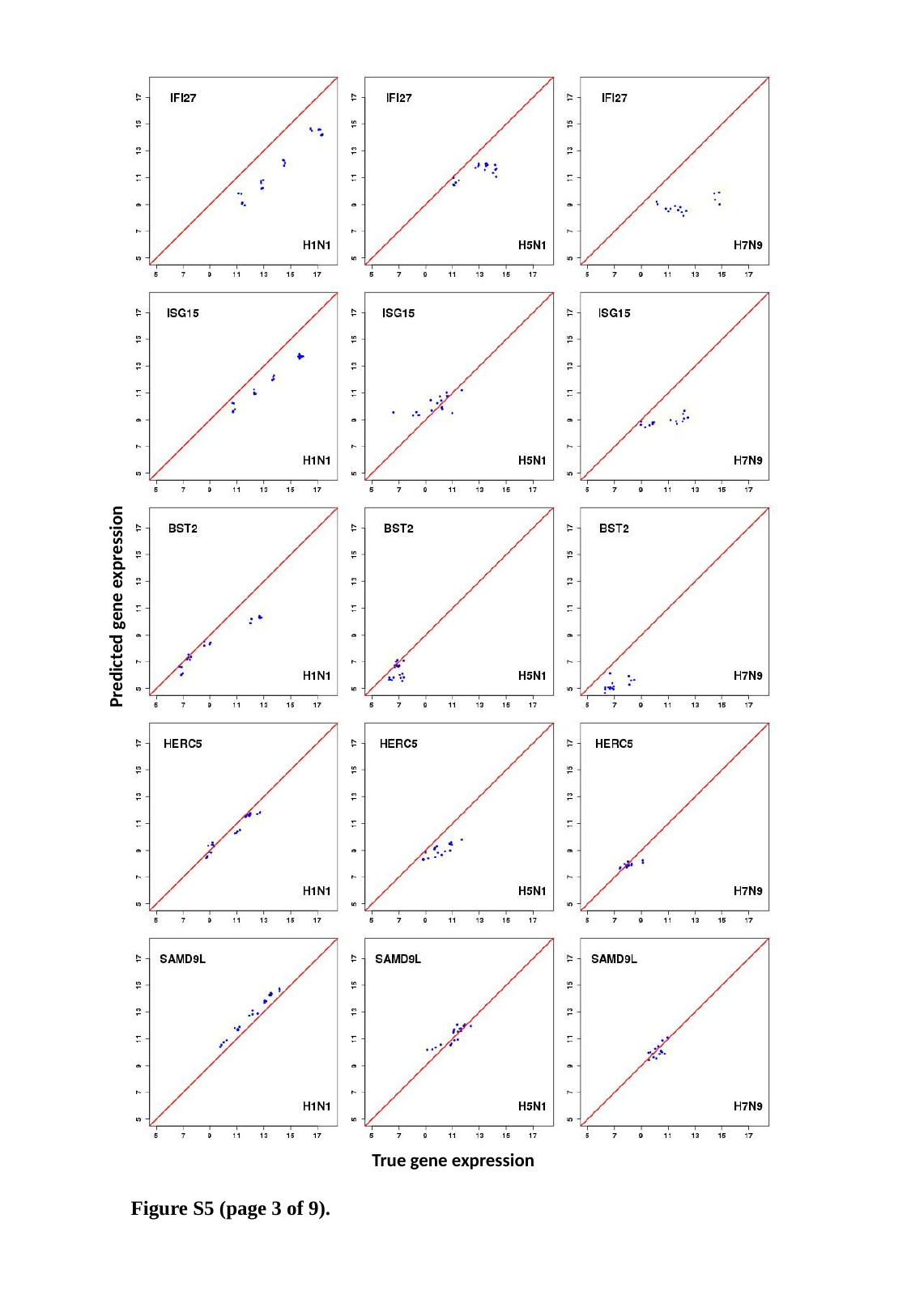

Predicted gene expression
True gene expression
Figure S5 (page 3 of 9).

## Slide 8
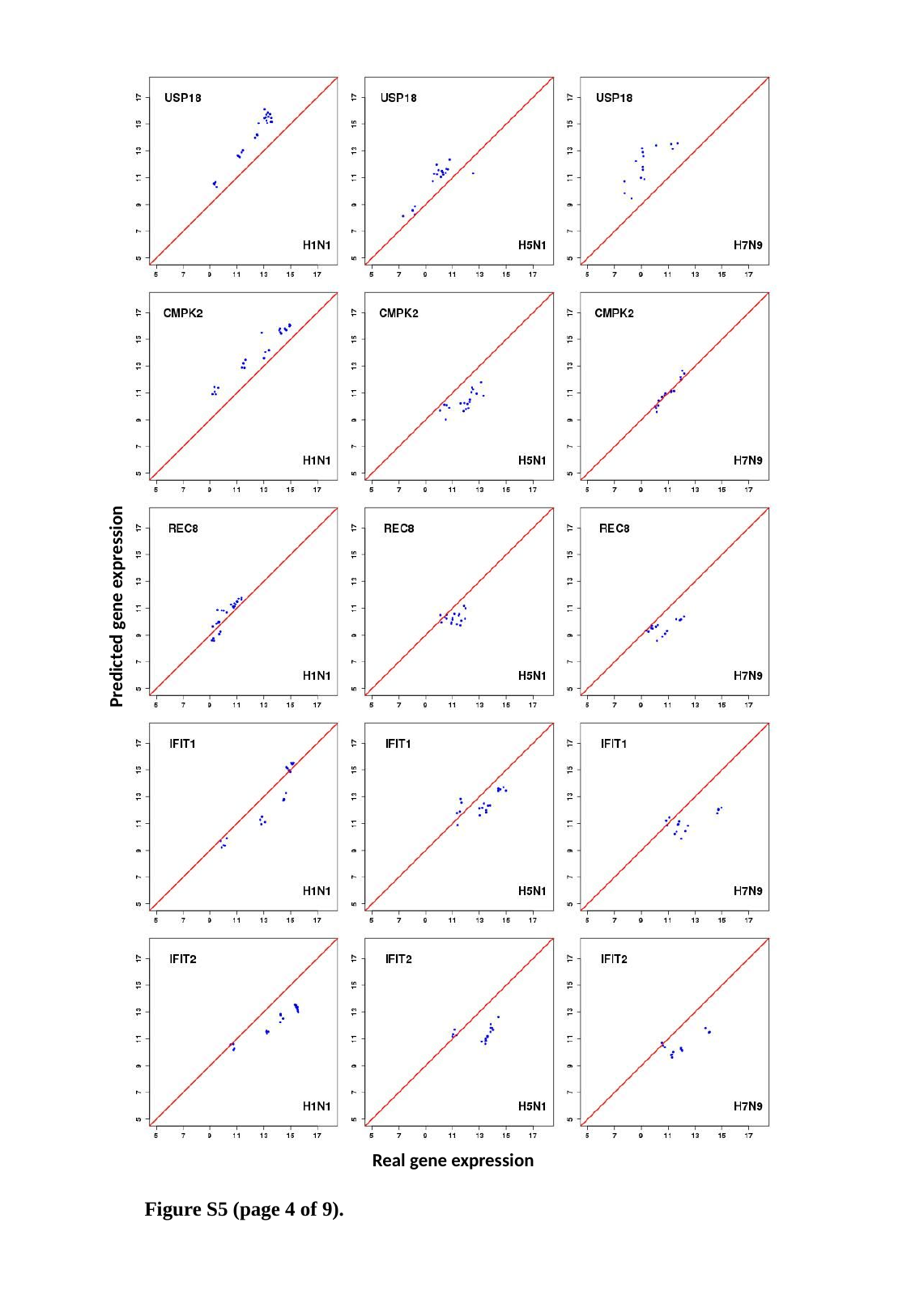

Predicted gene expression
Real gene expression
Figure S5 (page 4 of 9).

## Slide 9
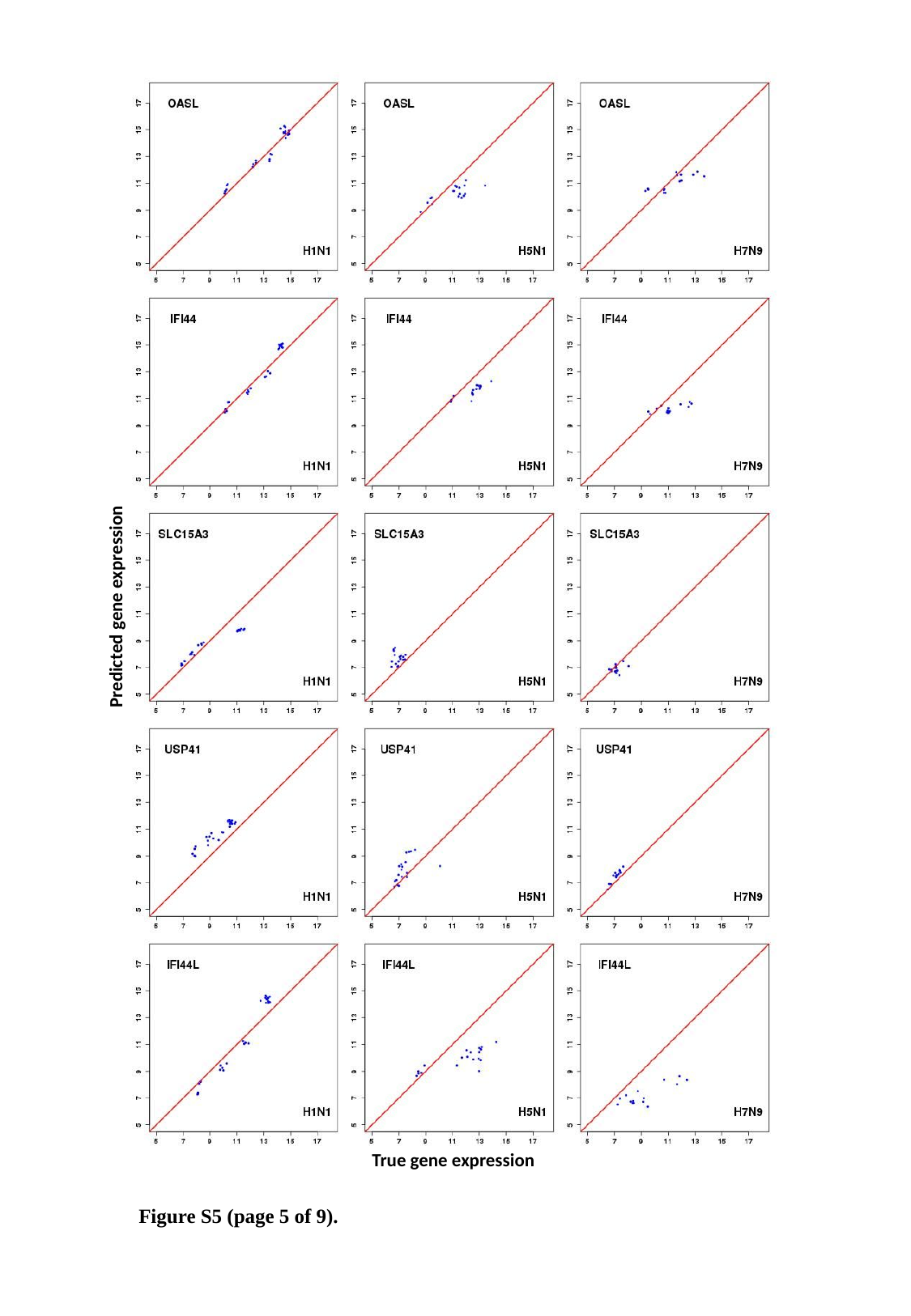

Predicted gene expression
True gene expression
Figure S5 (page 5 of 9).

## Slide 10
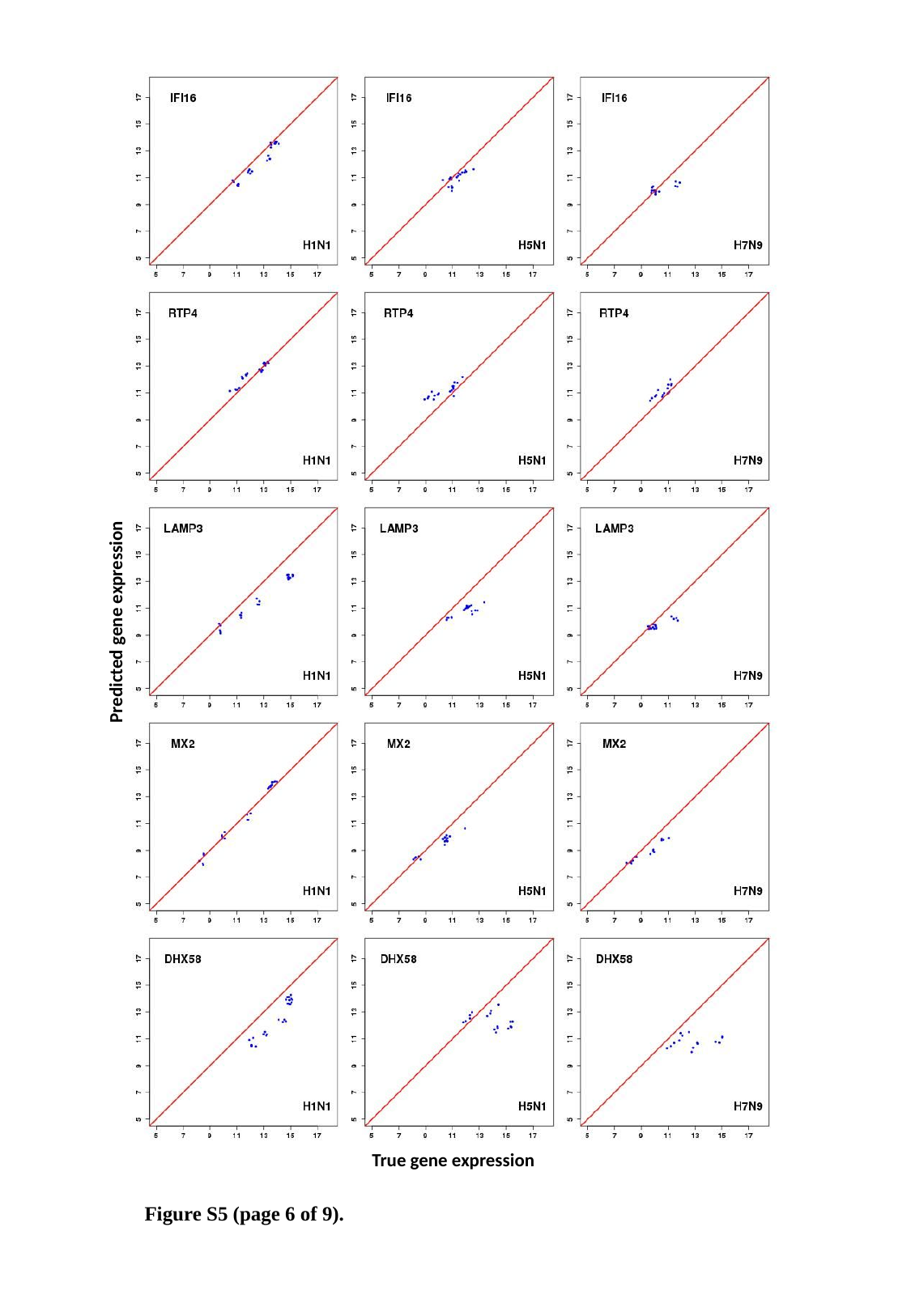

Predicted gene expression
True gene expression
Figure S5 (page 6 of 9).

## Slide 11
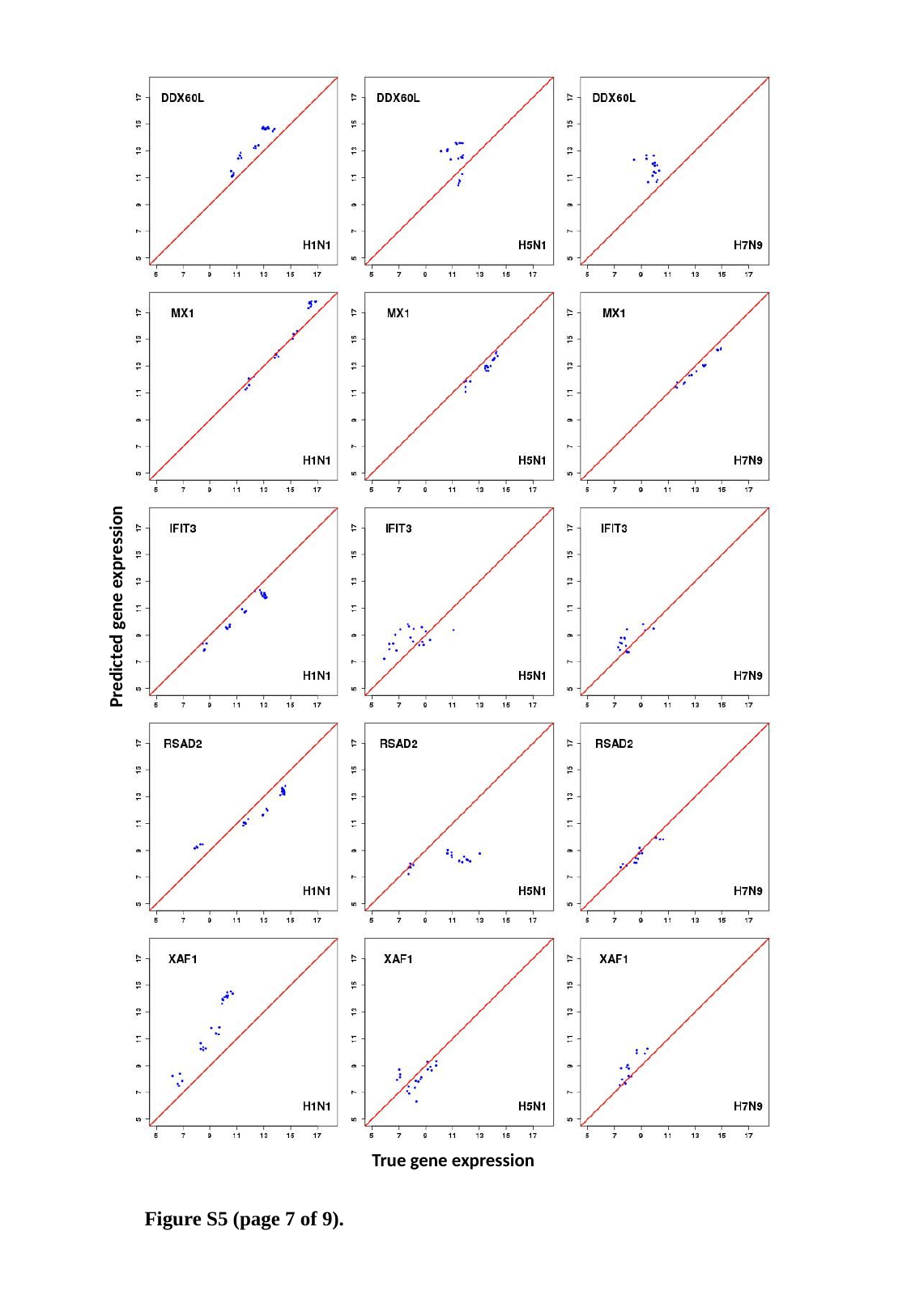

Predicted gene expression
True gene expression
Figure S5 (page 7 of 9).

## Slide 12
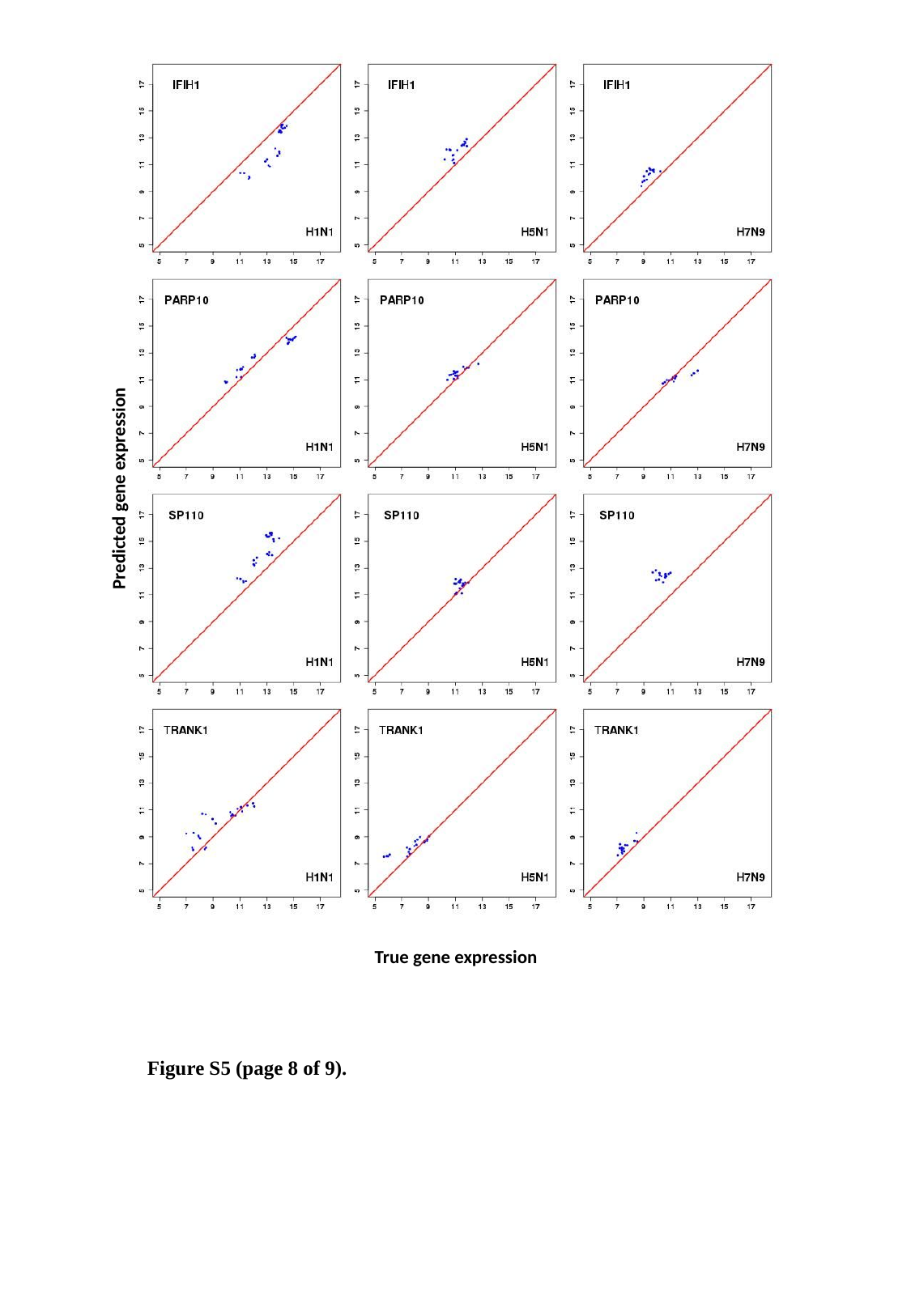

Predicted gene expression
True gene expression
Figure S5 (page 8 of 9).

## Slide 13
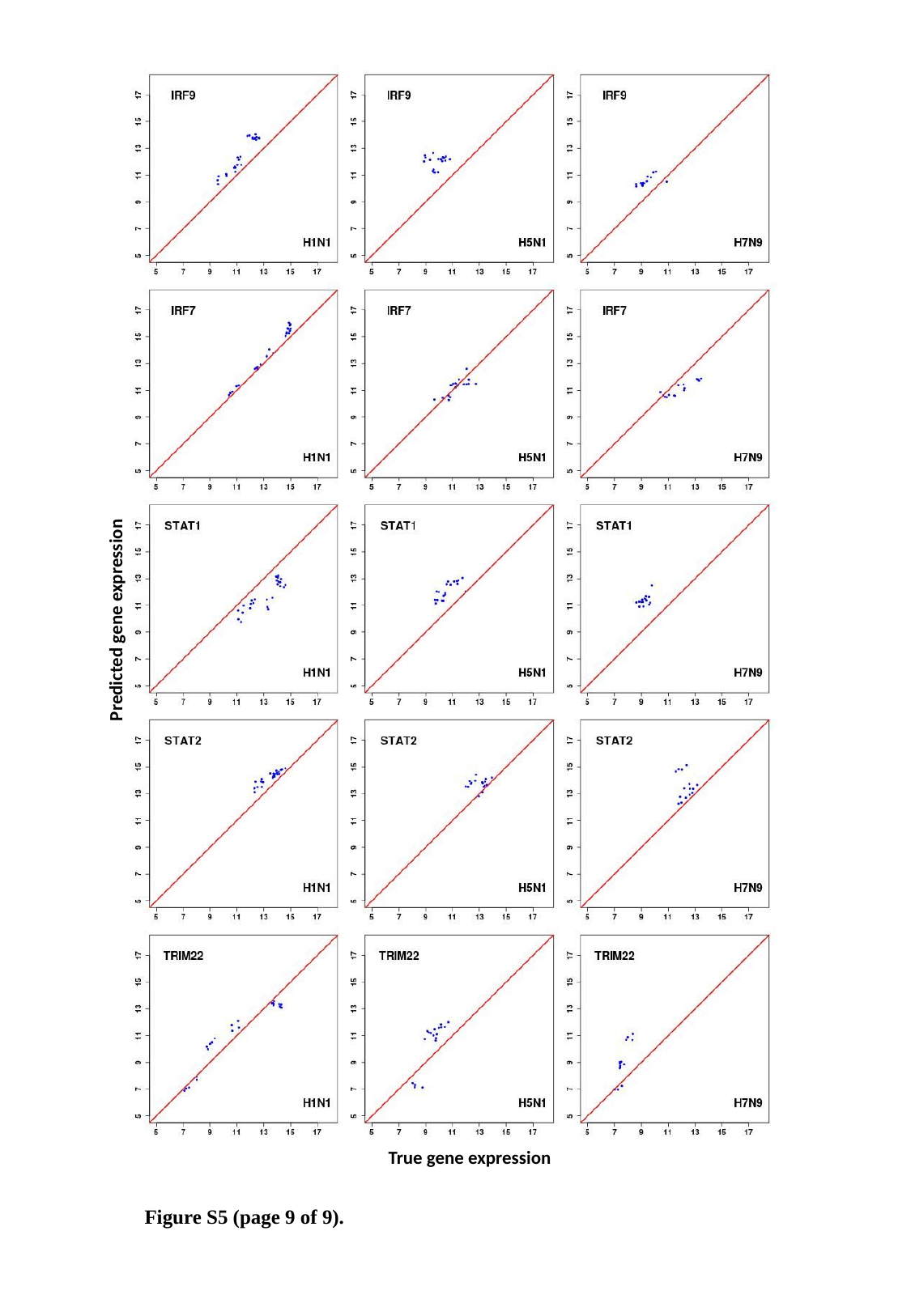

Predicted gene expression
True gene expression
Figure S5 (page 9 of 9).

## Slide 14
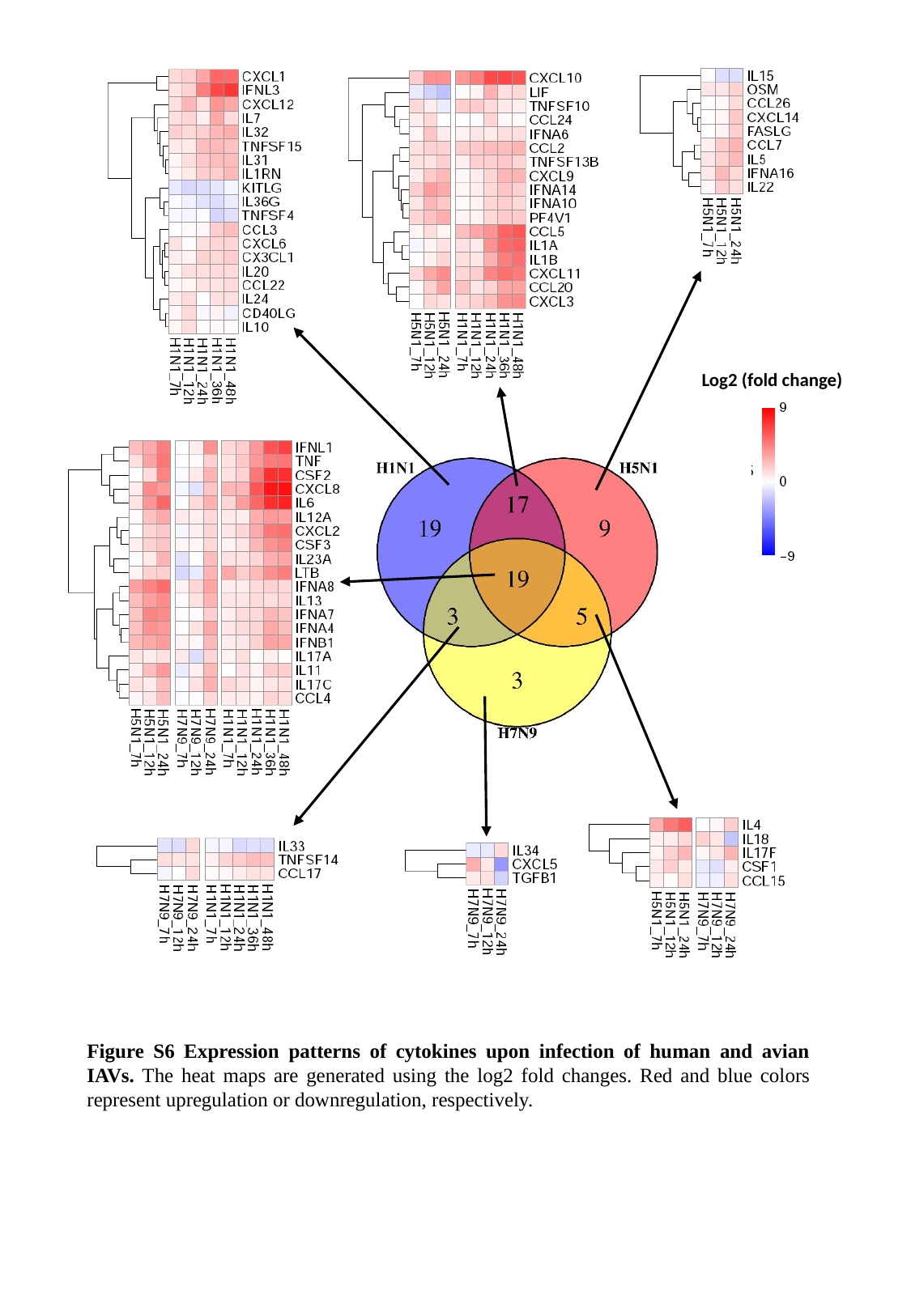

Log2 (fold change)
Figure S6 Expression patterns of cytokines upon infection of human and avian IAVs. The heat maps are generated using the log2 fold changes. Red and blue colors represent upregulation or downregulation, respectively.
